# APOL1 plasma membrane pools resist rapid protein degradation

**DOI:** 10.1038/s41598-026-37647-z

**Published:** 2026-02-16

**Authors:** Verena Höffken, Laura Alvermann, David Niggemeier, Katrin Beul, Pavel Nedvetsky, Bernhard Ellinger, Daria Assenmacher, Daniel Granado, Hermann Pavenstädt, Thomas Weide

**Affiliations:** 1 Medical Clinic D, University Hospital Münster, Albert Schweitzer-Campus 1, Geb. A14, 48149 Münster, Germany; 2 Department Screening Port, Fraunhofer Institute for Translational Medicine and Pharmacology ITMP, Schnackenburgallee 114, 22525 Hamburg, Germany

**Keywords:** APOL1, APOL2, AMKD, Degradation, Renal risk variants, surface-localized pools, IDR, Protein stability, Intracellular pools, Biochemistry, Cell biology, Computational biology and bioinformatics

## Abstract

Homozygosity of two sequence variants within the human *APOL1* gene, called *APOL1 G1* and *G2*, in combination with high Apolipoprotein L1 (APOL1) expression levels are linked to a wide spectrum of renal diseases summarized as APOL1-mediated kidney diseases (AMKDs). Previous studies have shown that inflammatory and immunomodulatory triggers are major contributors to elevated APOL1 protein expression. However, little is known about the stability of APOL1 and the role of protein degradation in regulating its intracellular levels. In this study, we systematically investigated these aspects. To investigate degradation dynamics, we used stable HEK293T cell lines with inducible overexpression of GFP-tagged APOL1 vA/G0, its C-terminal risk variants (G1, G2), N-terminal isoforms (vB1, vB3, vC), as well as the deletion mutant (ΔN59), APOL2, and an APOL1–APOL2 chimeric protein (NT_vA_-APOL2). Degradation and protein biosynthesis were modified using proteasome inhibitors and cycloheximide, respectively. Treated cells were analyzed using Western blotting, immunofluorescence microscopy and flow cytometry analyses. Moreover, *in silico* analyses were performed to identify motifs within the APOL1 sequence potentially mediating its degradation. This study shows that APOL1 is subject to remarkably rapid proteasomal degradation, observed for both the APOL1 wildtype (G0) and renal risk variants (RRVs) G1 and G2. Moreover, despite distinct topologies at the intracellular membranes of the APOL1 isoforms, all exhibit rapid protein degradation. In contrast, APOL2 – the closest homolog of APOL1 – demonstrated significantly greater resistance to proteasomal degradation. *In silico* analyses identified two intrinsically disordered regions (IDRs) present in APOL1 but absent in APOL2, potentially underlying the increased susceptibility to degradation. Notably, APOL1 surface-localized pools were resistant to rapid proteasomal degradation, with no major differences observed between G0 and RRVs. Together our findings suggest that APOL1 stability is highly compartment-specific, with rapid degradation at intracellular pools and pronounced stability at the cell surface. Targeting the stability of APOL1 at the PM represents a promising avenue for the development of novel therapeutic interventions against AMKD.

**Supplementary Information:**

The online version contains supplementary material available at 10.1038/s41598-026-37647-z.

## Introduction

In 2010, two independent studies revealed a strong association between kidney diseases and two sequence variants within the human *APOL1* gene, called *APOL1 G1* and *G2*, in comparison to the non-risk *G0* allele^1,2^. This correlation has initially been observed for HIV-associated nephropathy (HIVAN) and later also established for a wide spectrum of non-diabetic glomerular diseases, including hypertension-attributed kidney disease, primary focal segmental glomerulosclerosis , as well as lupus nephritis membranous nephropathy, and – as recently shown – also Covid-19 associated nephropathy (COVAN)^3–5^. To address how the increased genetic risk is linked to cellular injury — particular of podocytes — numerous model systems have been applied, including mouse^6–9^, fly (*Drosophila melanogaster*)^10,11^, *Xenopus*^12^, and zebrafish (*Danio rerio*)^13^ as *in vivo* systems, as well as the yeast *Saccharomyces cerevisiae*^14^ or various cell lines of human and non-human origin as single cell systems^1,15–19^.

These various approaches demonstrated the complexity of Apolipoprotein L1 (APOL1) intracellular effects and uncovered several potential pathomechanisms of APOL1-linked cytotoxicity, including apoptosis, autophagy-associated cell death, pyroptosis, disturbances of the ion homeostasis, as well as cellular exhaustion due to mitochondrial dysfunction and energy depletion^4,5^. A common pattern in most, if not all, of these *in vivo* and *in vitro* systems is a positive correlation between a trigger-induced increase of APOL1 expression and a subsequent increased level of APOL1-linked cytotoxicity, suggesting that the up-regulation of APOL1 expression above a critical level is the precondition for subsequent cellular damaging effects^20^.

The strong positive correlation between high APOL1 expression levels and cytotoxicity implies the existence of cellular mechanisms that tightly control the endogenous expression of APOL1 and/or its degradation or inactivation to prevent APOL1-induced cytotoxicity. However, whereas the factors that trigger APOL1 expression have been investigated and identified, less is known about the degradation of APOL1.

Here, we addressed this aspect by first investigating how APOL1 behaves compared to its closest evolutionary homolog APOL2. Next, we looked for potential differences in protein degradation between the wildtype and RRVs and between different APOL1 splice variants, which show different topologies, and between intracellular and surface-localized APOL1 pools.

Surprisingly, APOL1 underwent rapid degradation mainly via the proteasome, which appeared largely independent of risk and splice variants, whereas APOL2 was less susceptible to proteasome-dependent degradation. N-terminally modifications of APOL1 and APOL2 combined with degradation assays implied at least two regions regulating stability and turnover of APOL1. Using *in silico* tools, we identified two intrinsically disordered regions (IDRs), which were absent in APOL2, suggesting that they may be associated with the different degradation characteristics of APOL1 and APOL2. APOL1 surface pools, which are considered to contribute to APOL1-mediated cytotoxicity by forming a cation channel, are protected from rapid proteasomal degradation. Our data indicate that APOL1 integrated into the plasma membrane (PM) is more stable than APOL1 pools at intracellular membranes.

## Methods

### Constructs and cloning

Plasmids with the doxycycline (Dox) inducible pInducer21-puro expression system^21^ and GFP-tagged APOL variants used for transient transfections were already available and described elsewhere^15,22^. Constructs with inducible expression of APOL1 and APOL2 versions with C-terminal GFP and glycosylation tags^23^ were published before as well^22^. The newly generated constructs containing the N-terminal part (aa 1–59) of APOL1 fused to the APOL2 sequence (NT_vA_-APOL2) were cloned using the Gateway™ system. Insert was inserted into a pENTR GATEWAY™ vector by directional TOPO™ cloning using the pENTR™/D-TOPO^®^ Cloning Kit (K240020, Thermo Fisher) and shuttling into a pInducer21-puro expression vector using LR clonase™ enzyme mix II (11791100, Thermo Fisher). All used constructs possess the African haplotype “EIK”.

### Cell lines and cell culture

HEK293T cells were cultivated in cell dishes with Dulbecco’s modified eagle’s medium (DMEM, high glucose; D6429-500ML; Sigma-Aldrich) with 10% FCS and 1% Penicillin-Streptomycin (P0781-100ML, Sigma-Aldrich) at 37 °C and 5% CO_2_ atmosphere. Human immortalized podocyte cells (AB8, CIHP-1) were cultivated at 33 °C and 5% CO_2_ atmosphere in Roswell Park Memorial Institute medium (RPMI-1640, high glucose; R8758-500ML; Sigma-Aldrich) with 10% FCS (FBS.S 0615; BIO & SELL), supplements (2.5 ml HEPES (SH0887; Sigma-Aldrich), 0.5 ml NEAA (11140-035, Gibco), 0.5 ml Sodium pyruvate (S8636; Sigma-Aldrich), 0.5 ml insulin-transferrin-sodium solution (11074547001; Roche) and 1% Penicillin-Streptomycin. For induction of the overexpression in cells, 125 ng/ml Dox were added to the cultivation medium. Established stable HEK293T and AB8 cell lines inducibly expressing C-terminally GFP-tagged versions of APOL1-G0/vA, its C-terminal risk variants G1 or G2, previously described isoforms vB1, vB3, vC or the truncated ΔN59 as well as APOL2 were published elsewhere^17,22,24–26^. The newly stable HEK293T cell line with Dox-inducible expression of NT_vA_-APOL2-GFP and NT_vA_-APOL2-GFP-Glyc were generated using lentiviral transduction. Virus generation was performed in HEK293T cells transfected via jetOPTIMUS^®^ reagent (101000006, Satorius) with plasmid of choice and viral packaging plasmids and incubated over three days. Virus-containing supernatant was filtered through a 0.45 μm sterile filter, target cells (60–70% confluence) infected twice with a 1:1 ratio of virus suspension and fresh medium containing polybrene (8 µg/ml) for 24 h followed by regeneration phases of 24 h without virus. Afterwards, cells were selected by puromycin treatment (4 µg/ml). For inhibition of proteasomal activity, cells were treated with 5 µM MG132 (M7449-1ML, Sigma-Aldrich) or  100 nM Bortezomib (D4540, Sigma-Aldrich), for indicated time periods. Inhibition of protein biosynthesis was achieved by using 100 µg/ml Cycloheximide (CHX; C4859-1ML, Sigma-Aldrich).

### Transient transfections

For transient expression of constructs, cells were transfected with jetOPTIMUS^®^ reagent according to the manufacture’s instruction. In brief, for each well with 1 ml medium, 100 µl buffer, 1 µg DNA, and 1 µl reagent were mixed, incubated for 10 min at RT, and then added to cells at 70–80% confluency. For Dox-dependent expression, 1.25 ng/ml Dox were added to the medium immediately after transfection. After transfection and induction for 24 h, cells were washed with 1x phosphate-buffered saline (PBS) and processed for analysis.

### Preparation of cell lysates and PNGase F treatment

Cell lysate preparation and PNGase F treatment were performed as described earlier^22^. Briefly, for preparation of lysates, cells were grown on 12-well cell culture plates and washed with cold 1x PBS. For cell lysis, cells were transferred on ice and lysed in Lämmli buffer (20% (v/v) Glycerin, 125 mM Tris-HCl (pH 6.8), 10% (w/v) SDS, 0.2% (w/v) Bromphenole blue and 5% β-Mercaptoethanol in H_2_O) or RIPA buffer (50 mM Tris-HCl (pH 7.4), 150 mM NaCl, 1% NP-40, 0.5% Na-deoxycholate, 0.1% SDS) containing Complete protease inhibitor (04693116001; Roche) and phosphatase and protease inhibitor cocktails (P5726-5ML, P0044-5ML, P8340-1ML; Sigma-Aldrich). For homogenization, samples were passed ten times through a blunt 20-gauge needle (0.9 mm diameter), RIPA lysates were additionally sonicated for 10 min. Lämmli lysates were denatured by heating for 10 min at 95 °C, while RIPA lysates were centrifuged (20–30 min, 4 °C, 14,000 x g) to remove cell debris and nuclei. RIPA supernatants and Lämmli lysates were stored at -20 °C for further use. To determine APOL1 glycosylation status, RIPA lysates were digested with PNGase F to cleave off N-linked oligosaccharides (P0704L, New England Biolabs) according to the manufacturer’s instructions. Treated samples were analyzed via Western blot (WB).

### Western blot analyses

RIPA lysates were diluted in 2× Lämmli buffer and proteins were denatured (95 °C, 5 min). Proteins (10 µl per sample) were separated by 10% SDS PAGE (200 V, 45 min) and transferred by semi-dry blotting onto a PVDF membrane (0.45 μm pore size; IPVH00010; Merck Millipore, Burlington, MA, USA) activated with methanol for 90 min at 1 mA/cm^3^. Membranes were blocked with 5% milk blocking (T145.2, Roth) in TBST for 30 min and incubated with rabbit anti-APOL1 antibody (1:1000; HPA0018885, Sigma), rabbit anti-APOL2 antibody (1:1000; HPA001078, Sigma), or rabbit anti-α-Actinin4 antibody (1:750; ALX-210-356, Enzo; or 1:1000; S15145S, Cell Signaling) diluted in 5% bovine serum albumin (BSA; 8076.3, Roth) in TBST overnight at 4 °C. After washing membranes three times in TBST, secondary horseradish peroxidase-coupled goat anti-mouse IgG antibody (1:2000; 115-035-068; Jackson ImmunoResearch) or goat anti-rabbit IgG antibody (1:2000; 115-035-144; Jackson ImmunoResearch) diluted in blocking solution was added for 1 h at RT. Unbound antibody was removed by washing with TBST three times before detection of proteins with Clarity™ Western ECL substrate (1705061; BioRad) on an Azure c600 Compare cSeries imaging system (Azure Biosystems Inc., Dublin, CA, USA).

### Immunofluorescence (IF) and live cell microscopy

Staining of APOL1 at the PM was performed as described elsewhere^26,27^. In brief, 24 h induced cells on coverslips were fixed in 4% paraformaldehyde (PFA; P087.1, Roth) for 15 min. After carefully washing with 1x PBS twice for 10 min, unpermeabilized cells were incubated for 1 h with primary rabbit anti-APOL1 antibody (11486-2-AP, ProteinTech) diluted 1:200 in 1x PBS on ice. After washing in 1x PBS two times, cells were incubated with secondary fluorophore-coupled goat anti-rabbit-AlexaFluor^®^647 antibody (AF647, A-21244, Invitrogen) diluted 1:1500 in 1x PBS for 1 h on ice. Subsequently to the counterstaining of DNA with DAPI (1:5000), cells were washed and mounted in Mowiol. Samples were analyzed using a fluorescence microscope (Axio Observer Z1, HXP120, Axiocam MRm; Carl Zeiss) with EC Plan-Neofluar 63x objective using immersion oil (Zeiss) and corresponding Zen Blue software (v2.3).

For live cell microscopy, APOL-GFP AB8 cells were seeded in 8-well chamber slides (94.6190.802; Sarstedt) and Dox-induced for 24 h. Nuclei were counterstained with Hoechst33342 (1:4000; Thermo Fisher) for 20 min, MG132 treatment was performed as described before. Cells were imaged live with a Leica SP8 laser scanning confocal microscope and HC PL APO 63x/1.40 OIL CS2 objective and the corresponding LASX software.

### Flow cytometry (FC)

HEK293T cells were grown and treated on 12-well cultivation plates before preparation for FC analysis. After washing cells twice with 1x PBS, cells were resuspended in 500 µl 1x PBS and transferred into round bottom tubes. After centrifugation for 5 min at 1000 x g supernatant was discarded, cells were resuspended in 400 µl 1% PFA in PBS and stored dark at 4 °C until measurement. For analysis of the APOL1 surface level^26,27^, the staining was performed similarly as for microscopy analyses. Briefly, cells were fixed by resuspending with 2% PFA (15 min, RT), before centrifuged at 1000 x g at 4 °C for 5 min. After washing twice with 2 ml cold PBS, cells were resuspended and incubated in 100 µl primary rabbit anti-APOL1 antibody (11486-2-AP, ProteinTech) diluted 1:200 in PBS for 1 h on ice. After a double washing and centrifugation step, cells were incubated for 1 h on ice in 100 µl secondary fluorophore-coupled goat anti-rabbit AF647 diluted 1:1500 in PBS. After washing with PBS, cells were resuspended in 400 µl PBS and stored at 4 °C in the dark until measurement.

FC cytometry analysis was performed using a FACSCalibur™ Flow cytometer (BD Biosciences) using a blue argon laser (488 nm) and a red diode laser (635 nm) with detectors for forward scatter (FSC, determination of cell size), sideward scatter (SSC, determination of internal complexity e.g. granularity) and fluorescent channels according to GFP signal (515–545 nm) or AF647 signal (653–669 nm). Detection was done using the corresponding CellQuest Pro software. For FACS measurement of GFP intensity in degradation experiments, 20 000 viable cells were measured. For measurement of GFP and AF647 intensities of cells with stained surface APOL1, 50 000 viable cells were measured, while for transient transfection experiments, GFP and AF647 intensities were measured of 100 000 viable cells.

For FC data analysis FlowJo (v10.10.0) was used. Cells were gated for viable cell population with a FSC height > 170. Subsequently, GFP negative signal was defined using the 95th percentile of GFP intensity of the viable cells in uninduced condition (-Dox) per stable cell line or of the viable HEK293T wildtype control in transient transfections. This gate was transferred to induced cells to define the positive induced GFP signal representing the APOL-GFP expression (Suppl. Fig. SF4). For surface stained APOL1, specific detection of secondary antibody was tested using a “no stain” control, lacking the incubation with the primary antibody. Unspecific signals of surface APOL1 staining were identified by gating the 95th percentile of the viable HEK293T wildtype control. Similarly to GFP determination, the gate was applied to the other conditions to determine the AF647 positive signal (Suppl. Fig. SF4). GFP and AF647 gates were combined to define quartiles Q1-Q4.

### Cell viability assays

To address the cell viability in real time, the RealTime-Glo™ Cell Viability Assay (G9712, Promega) according to the manufacturer’s instructions. In brief, 10 000 HEK293T cells per well were seeded in 50 µl cultivation medium onto 96-well white bottom plates (Nunclon delta; 136101, ThermoFisher Scientific). After 16–18 h incubation time, 50 µl 2x RealTime-Glo™ enzyme-substrate mix (1:1) were diluted in 50 µl medium and added to each well either with or without 125 ng/ml Dox^15^. Luminescence was measured at indicated time points using a microplate reader (TECAN). For each cell line, at least three independent experiments (*N* = 3), including six technical replicates for each N (*n* = 6), were measured.

### *In Silico* prediction of intrinsically disordered regions (IDRs)

Identification of IDRs within APOL protein sequences *in silico*, we applied five different IDR prediction tools: ADOPT (https://adopt.peptone.io/)^28^, DisEMBL™ (http://dis.embl.de/)^29^, (https://biomine.cs.vcu.edu/servers/flDPnn/)^30^, IUPred2A (https://iupred2a.elte.hu/)^31,32^, and the artificial network supported version AIUPred (https://aiupred.elte.hu/)^33^ using standard settings and recommended threshold for interpretation. Disordered regions were identified according to program specific scores (ADOPT: Z score < 8 an < 3; DisEMBL: by LOOPS/COIL definition; flDPnn: propensity < 0.3; IUPred2A, AIUPred and PrDOS: confidence scores < 0.5) and subsequently filtered for a continuous length of ≥ 15 aa and appearance in at least two algorithms. PEST prediction was performed using EMBOSS program epestfind (https://emboss.bioinformatics.nl/cgi-bin/emboss/epestfind), but no potential PEST motifs could be identified within APOL1 or APOL2.

### Statistical analyses and figure Preparation

Statistical analyses were performed using the 1-way ANOVA with Dunnett’s test for multiple comparison if not stated otherwise. Significance was calculated between controls (-Dox or HEK293T ctr) and treated samples, and asterisks indicate the level of statistical significance of an adjusted p value: * *p* < 0.05, ** *p* < 0.01, *** *p* < 0.001 and **** *p* < 0.0001. Image files were further processed using ImageJ and FIJI. Data are presented as means with SEM. Graphs and statistics were generated using GraphPad Prism (v10.4.2). Figures were prepared using Adobe Illustrator (v15.0.0). Schematic illustrations were generated using BioRender (https://BioRender.com).

## Results

### APOL1 is predominantly degraded via the proteasome

**Fig. 1:**
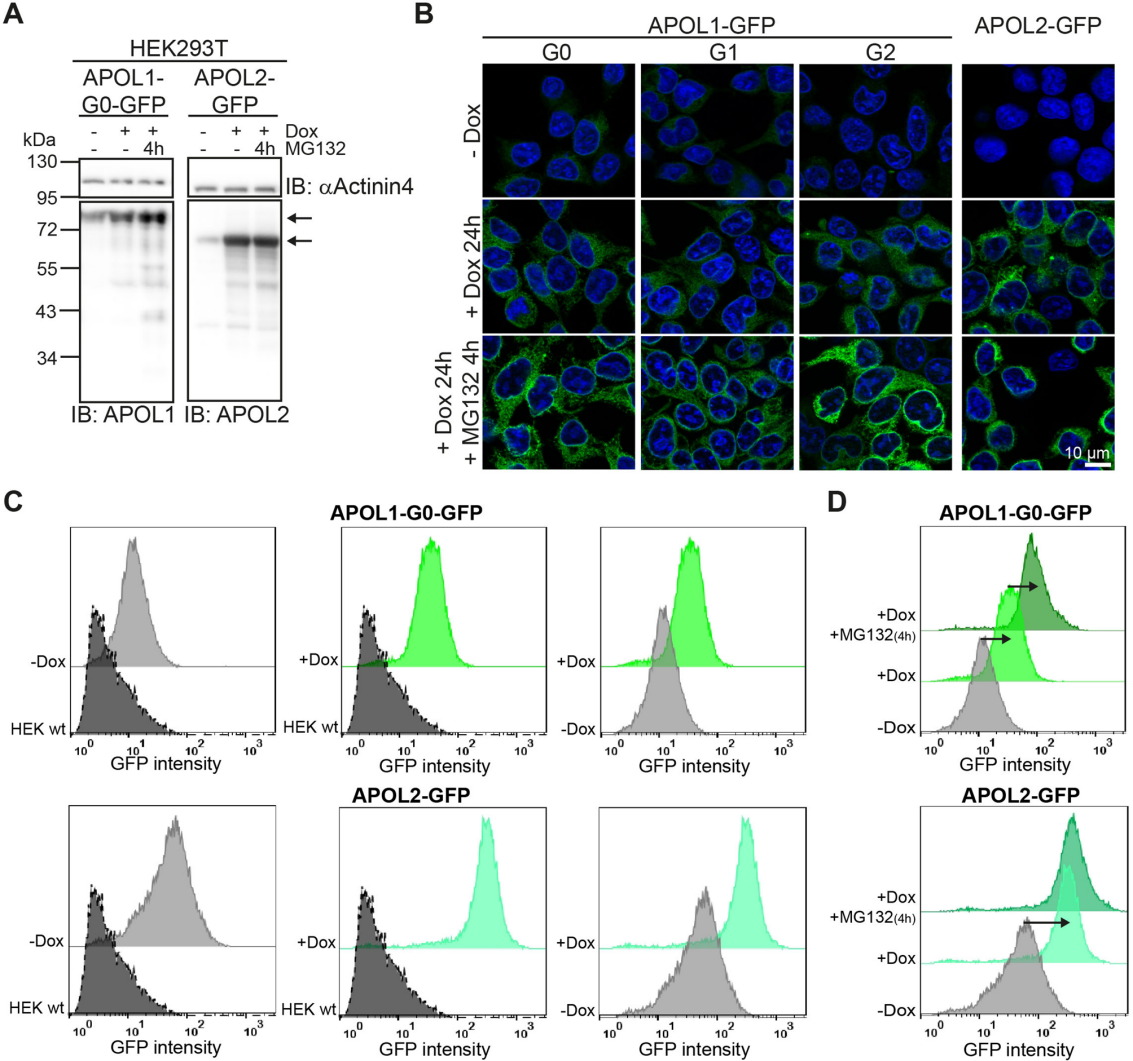
APOL1 is degraded via the proteasome. HEK293T cell lines enabling a doxycycline-dependent expression of C-terminally GFP-tagged APOL1 wildtype (G0) and APOL2 were treated with proteasome inhibitor MG132. (**A**) WB analysis of non-induced (-Dox), induced (+ Dox) and MG132 treated HEK293T expressing APOL1 G0 and APOL2. α-Actinin4 served as loading control. (**B**) IF images (overview and details) of HEK293T expressing APOL1 G0, the risk variants G1 and G2, and APOL2. *Upper panel*: non-induced cells (-Dox). *Middle panel*: APOL1 and APOL2 cells in which expressions were triggered with 125 ng/ml doxycycline for 24 h (+ Dox). *Lower panel*: Dox-induced cells with an additional MG132 treatment (5 µM) for 24 h (+ Dox, +MG132). (**C**) Histogram of flow cytometric (FC) analyses of APOL1 (G0) and APOL2: *Left*: non-transduced HEK293T control cells (dark grey) *versus* non-induced APOL1 G0 (-Dox) cells (light grey). *Middle*: control (dark grey) cells versus doxycycline-induced APOL1 (green) or APOL2 (turquoise). Right: non-induced cells (light grey) versus doxycycline-induced (+ Dox) APOL1 (green) and APOL2 (turquoise) cells. (**D**) Representative composed histogram: FC analysis showing APOL1 G0 and APOL2 cells that were non-induced (-Dox), induced (+ Dox), or induced with an additional MG132 treatment (+ Dox +MG132) for 4 h in APOL1 (dark green) or APOL2 (dark turquoise). (*N* ≥ 3); y-axis: cell count; x-axis: GFP fluorescence.

Previous studies showed that APOL1 cytotoxicity is dose- and variant-dependent. Here, we investigate how degradation can contribute to APOL1 expression levels of the main splice variant A (African haplotype “EIK”). To address this aspect, we induced the expression of C-terminally tagged APOL1 G0 and RRVs G1 and G2 of the main variant A, and APOL2 in HEK293T cell lines with doxycycline (Dox)^15,22^ in the absence or presence of the proteasome inhibitor MG132. In line with previous results, we observed a robust expression of APOL1 and APOL2 GFP fusion proteins after induction by Dox in WB analyses (Fig. 1A) and immunofluorescence imaging experiments (Fig. 1B). Strikingly, MG132 treatment results in an additional increase in the signal intensity for APOL1 G0 and RRVs, but not for APOL2 (Fig. 1B), suggesting that proteasomal inhibition enhances expression levels of APOL1, but not of APOL2. An analogous approach using immortalized podocytes in live cell imaging showed similar results and confirmed the differences between APOL1 and APOL2 (Suppl. Fig. SF1). To validate these data, we performed flow cytometry (FC), as it allows quantitative analysis of a large number of cells (Fig. 1C). Non-transduced HEK293T cells served as reference (negative control). FC analyses showed that stable cells lines possessed a background expression of APOL1 or APOL2-GFP fusion protein even in the absence of induction (-Dox). Doxycycline administration to the cells (+ Dox) resulted in a significant increase in expression levels (Fig. 1C). Inhibition of the proteasome by MG132 resulted in a further intensity increase of APOL1-G0-GFP expressing cells. The distribution of cells expressing APOL2-GFP remained unchanged upon MG132 treatment (Fig. 1D). Together, these data show that the blockade of the proteasome increases expression levels of APOL1 G0 and RRVs, but not of APOL2.

### APOL1 and APOL2 show different degradation kinetics

**Fig. 2:**
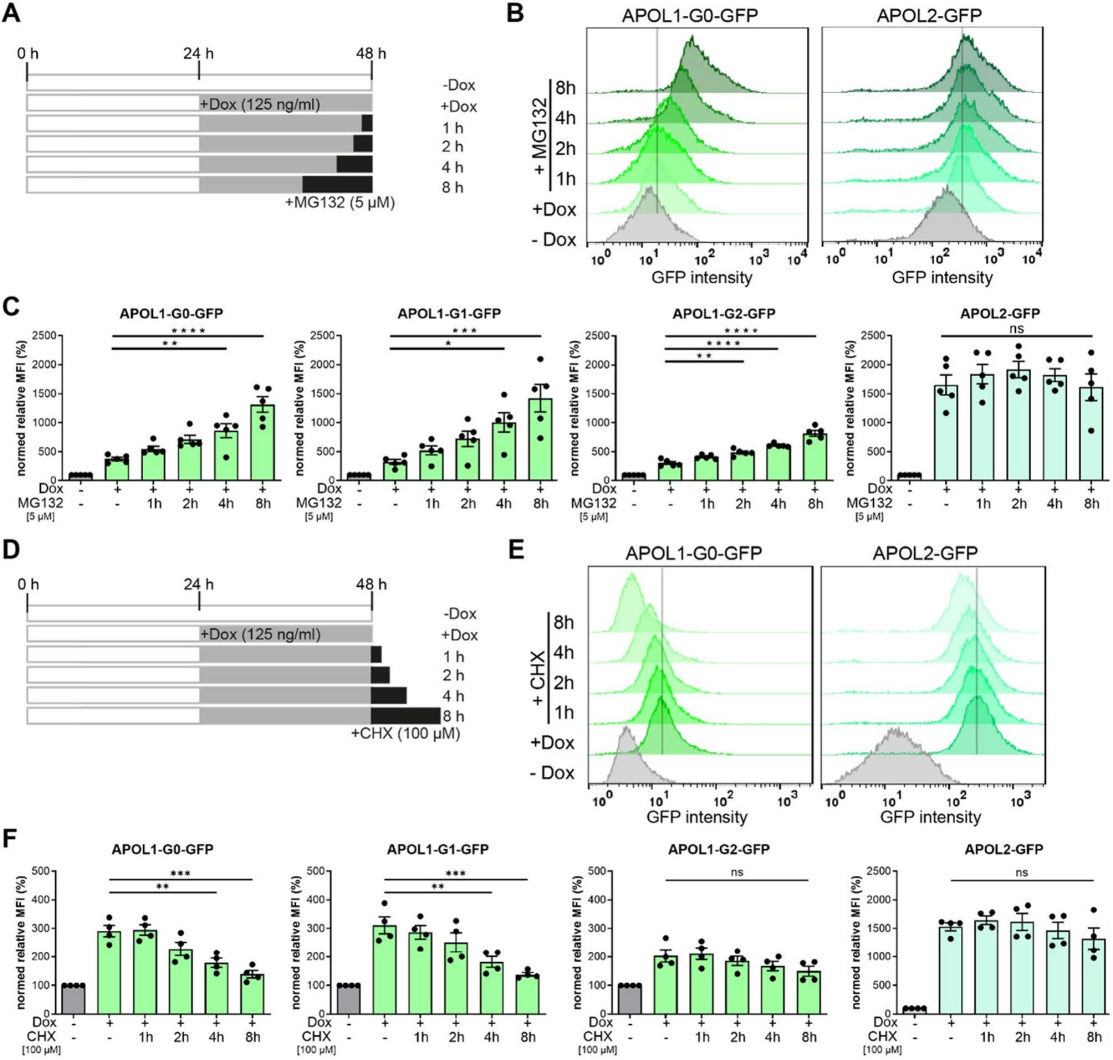
APOL1 and APOL2 show different degradation dynamics. (**A**) Experimental setup (scheme): HEK293T cells expressing C-terminally GFP-tagged APOL1-G0 and APOL2 were induced for 24 h with doxycycline, with or without MG132 treatment (5 µM). (**B**) Representative composed histogram of FC analyses with non-induced (-Dox) and induced (+ Dox) APOL1 G0-GFP *(left)* and APOL2-GFP-expressing cell lines *(right)* with or without proteasomal inhibition by MG132 for different periods (*N* ≥ 3); y-axis: cell count; x-axis: GFP fluorescence. (**C**) *Graphs*: FC analyses of non-induced, induced APOL1 (G0, and RRVs in green) and APOL2 (turquoise) cells with or without MG132 treatment summarized using the mean fluorescence intensity (MFI). (**D**) Experimental setup (scheme): HEK293T cells expressing C-terminally GFP-tagged APOL1-G0 and APOL2 were induced for 24 h with doxycycline, with or without inhibition CHX treatment (100 µM). (**E**) Composed histogram of FC analyses with non-induced (-Dox), induced (+ Dox) APOL1 G0-GFP *(left)* and APOL2-GFP-expressing cell lines *(right)* with or without inhibition of the biosynthesis by CHX for different periods (*N* ≥ 3); y-axis: cell count; x-axis: GFP fluorescence. (**F**) *Graphs*: FC analyses of non-induced, induced APOL1 (G0, and RRVs in green) and APOL2 (turquoise) cells with or without CHX treatment summarized using the mean fluorescence intensity (MFI). ns: not significant, *: *p* < 0.05, **: *p* < 0.01***: *p* < 0.001, ****: *p* < 0.0001.

Next, we aimed to determine if there are differences in the degradation kinetics between APOL1 G0 and the renal risk variants. Therefore, cell lines allowing conditional expression of APOL1-GFP forms G0 and RRVs as well as APOL2-GFP (Fig. 1) were incubated with proteasomal degradation inhibitors (MG132 and Bortezomib) for different time periods (Fig. 2A). FC measurements followed by data analyses using the GFP fluorescence signal as the readout, showed a continuous increase in the GFP fluorescence signal within eight hours for APOL1, but not for APOL2 in the presence of proteasome inhibitors (Fig. 2B). A rapid increase of GFP signal also occurred for RRVs G1 and G2 under Bortezomib (a clinically approved proteasome inhibitor) treatments, while APOL2 level was much less affected (Fig. 2C, Suppl. Fig. SF2A).

Cycloheximide (CHX) is an inhibitor of eukaryotic protein biosynthesis that blocks the elongation step of translation by interfering with the ribosome. This prevents the addition of amino acids to the growing polypeptide chain. We hypothesized that inhibition of protein biosynthesis by CHX would lead to a rapid decrease in the levels of short-lived/unstable APOL1, while the effect on more stable APOL2 will be less pronounced. This approach may also help to uncover potential differences in the stability of RRVs compared to G0. To test this, we induced the expression of APOL1- and APOL2-GFP fusion proteins for 24 h and then added CHX to the cells for different time periods (Fig. 2D). The FC studies and WB experiments demonstrate a rapid decrease in APOL1 G0 level within eight hours (Fig. 2E, Suppl. Fig. SF2B). The strong decrease was also evident for the RRVs, G1 and G2 (Fig. 2F). In contrast, the expression levels of APOL2 remained nearly constant in the presence of CHX treatment during this period (Fig. 2E, F). Thus, the experiments show that, first, APOL2 is more stable and resistant against proteasomal degradation than APOL1, and secondly that the RRVs do not differ in their degradation dynamics from the G0 variant of APOL1.

### Distinct APOL1 isoforms display similar degradation kinetics

**Fig. 3:**
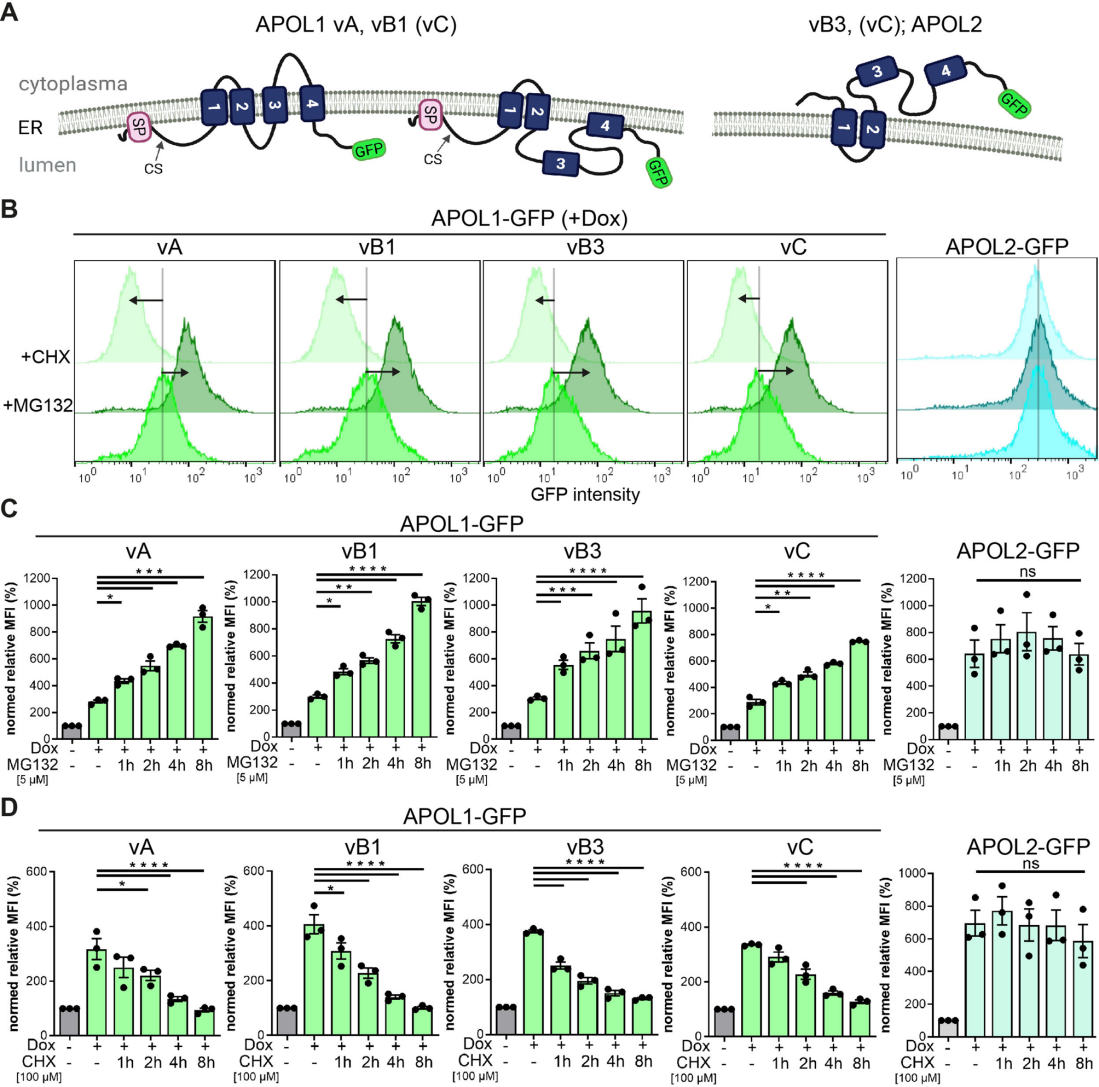
Rapid APOL1 degradation is isoform-independent. HEK293T cells allowing the conditional expression of C-terminal GFP-tagged APOL1 splice variants (G0) with and without MG132 treatment. (**A**) Scheme: Membrane orientation of APOL1 isoforms. *Left*: APOL1 isoforms vA, and vB1 (vC) with N- and C-termini facing the ER lumen. *Right*: APOL1 isoform vB3 and APOL2 with both termini on the cytoplasmic side. (**B**) Composed histograms of FC analyses with APOL1 G0-GFP isoforms vA, vB1, vB3 and vC expressing cells without further treatment (+ Dox), or in combination with MG132 or cycloheximide (CHX) respectively. The histograms show representative FC analyses of at least three independent experiments (*N* ≥ 3); y-axis: cell count; x-axis: GFP-fluorescence. (**C**,**D**) *Graphs*: FC analyses of the MG132 (**C**) and CHX (**D**) treatments shown as MFI values. -Dox: non-induced cells; +Dox: induced for 24 h without inhibitor and with MG132 or CHX treatment for indicated time periods (*N* ≥ 3); y-axis: cell count; x-axis: GFP-fluorescence. ns: not significant, *: *p* < 0.05, **: *p* < 0.01, ***: *p* < 0.001, ****: *p* < 0.0001.

Previous studies have demonstrated that the *APOL1* gene is alternatively spliced, resulting into different isoforms^17,24,25,34^. These APOL1 isoforms differ in their orientation (topology) within membranes^17,22,24^. While in isoforms with a functional signal peptide (vA, vB1, vC) both termini face the same luminal direction, the N- and C-termini of vB3 and APOL2 lacking signal peptides are oriented contrarily^22,26^. Moreover, this signal peptide also defines the presence of APOL1 at the PM (and in the serum). In contrast to the primary splice variant vA (and to vB1 and vC), vB3 exhibits a distinct topology at the ER membranes, which is similar to the APOL2 topology^22^. This suggests that vB3, due to its unique APOL2-like topology, may display different degradation dynamics compared to the other APOL1 splice variants (Fig. 3A). To test this, we expressed APOL1 G0 splice variants as GFP fusion proteins and performed MG132 inhibitor studies, as described above and analyzed the cells by FC. Strikingly, following MG132 treatment, all APOL1 isoforms, including APOL1 vB3, showed a similar accumulation pattern over time. In all cases, accumulation began within the first hour and increased stepwise over the subsequent eight hours of MG132 treatment, whereas APOL2 expression levels remained nearly constant throughout the period (Fig. 3B, C). Inhibition of protein biosynthesis via CHX led to a rapid decrease in expression levels of all APOL1 isoforms, but not of APOL2 (Fig. 3B, D), thus confirming an isoform-independent rapid degradation of APOL1. Together, these experiments demonstrate that rapid APOL1 degradation occurs independent of the APOL1 topology at cellular membranes.

### The N-terminal region of APOL1 contributes to proteasomal degradation

**Fig. 4:**
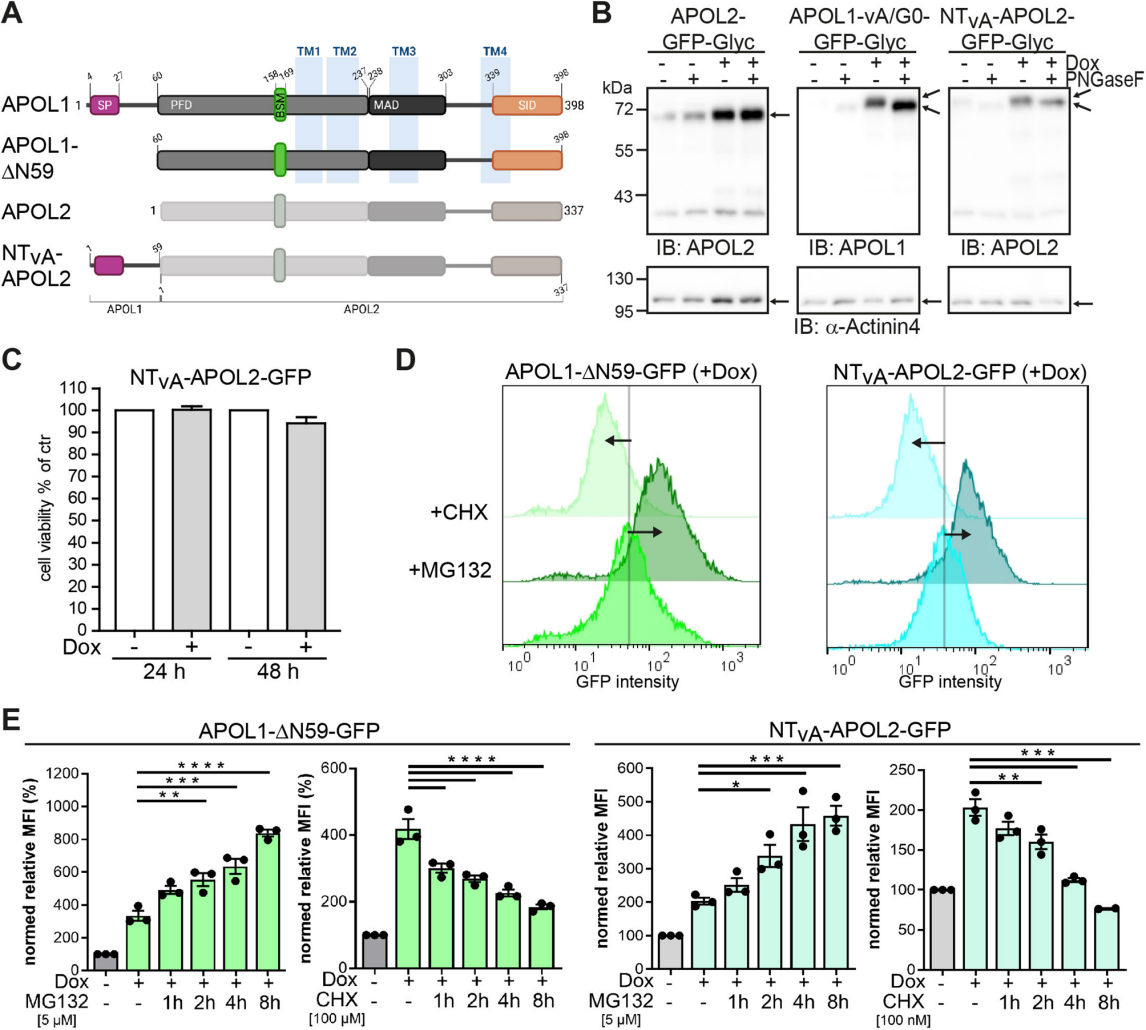
The N-terminal region of APOL1 contributes to proteasomal degradation. (**A**) Scheme of used APOL1-APOL2 fusion and deletion mutants. Indicated are the signal peptide (SP), putative transmembrane helices (TM) and PFD, MAD and SID regions within the APOL1 and APOL2 primary structure. (**B**) Determination of NT_vA_-APOL2 membrane topology. WB analyses of lysates from cells expressing vA APOL1 *(left)*, APOL2 *(middle)* and the NT_vA_-APOL2 *(right)* fused to a combined GFP-Glyc-tag (Glyc) with and without PNGase F treatment. α-Actinin-4 served as loading control. (**C**) Viability assay of cells expressing GFP-tagged NT_vA_-APOL2 (*N* = 3, *n* = 6). (**D**) Composed histograms of FC analyses of APOL1 G0-GFP splice variants (vA, vB1, vB3 and vC) expressing cells with induction (+ Dox), and in combination with MG132 or cycloheximide (CHX) respectively. (**E**) *Graphs*: FC analyses of APOL1 ΔN59 and NT_vA_-APOL2 expressing cells in combination with MG132 and CHX treatments shown as MFI values. -Dox: non-induced cells; +Dox: induced for 24 h without inhibitor or with MG132 (+ Dox +MG132) or CHX treatment (+ Dox +CHX) for indicated time periods. The graphs show data of at least three independent experiments (*N* ≥ 3; y-axis: cell count; x-axis: GFP fluorescence. ns: not significant, *: *p* < 0.05, **: *p* < 0.01, ***: *p* < 0.001, ****: *p* < 0.0001.

From an evolutionary perspective, APOL1 and APOL2 are the closest relatives and show a high degree of homology in their sequences^22^. However, in contrast to APOL2, APOL1 contains an N-terminal extension, which is present in all APOL1 splice isoforms, even in isoform vB3 that lacks a functional signal peptide^17,22^. To test whether this APOL1-specific N-terminal region contributes to its rapid degradation we included two further proteins into our studies: first, an APOL1 truncation mutant (APOL1 ΔN59), lacking the complete N-terminal region (amino acids 1–59 of vA), and second, a protein in which these 59 aa of the N-terminus of APOL1 vA were fused to APOL2 (NT_vA_-APOL2, Fig. 4A).

To analyze the topology of these proteins we fused an artificial N-glycosylation tag (Glyc-tag^23^, to their C-terminal ends. Expression of these proteins with subsequent PNGase F treatment allows investigating whether the C-termini were inside the ER lumen – making them accessible to N-glycosylation – or if they are facing the cytoplasm. Previously, these Glyc-tag-based approaches showed that the truncation of APOL1’s first 59 aa results in a topology switch in which the N- and C-terminal regions face the cytoplasm, like APOL2, whereas all other APOL1 splice variants (vA, vB1 and vC) carry their C-termini inside the ER lumen^22^. The Glyc-tag assays revealed that transferring the first 59 amino acids of APOL1 to APOL2 induced a topology switch, positioning both the N- and C-termini inside the ER lumen (Fig. 4B), without altering the non-toxic properties of APOL2 (Fig. 4C). Treatment of cells expressing either the APOL1 truncation mutant ΔN59 or the NT_vA_-APOL2 chimera with MG132 and CHX revealed that the NT_vA_-APOL2 chimera adopts degradation dynamics resembling those of APOL1 rather than APOL2, whereas ΔN59, interestingly, maintains a degradation pattern characteristic of all APOL1 risk and splice variants (Fig. 4D, E). This indicates that the N-terminal region contains signals responsible for accelerated degradation of APOL1 and can destabilize APOL2 if fused to it. However, rapid disappearance of the APOL1 ΔN59 mutant suggests additional sequences regulating APOL1 stability and degradation.

### APOL1 contains two putative intrinsically disordered regions (IDRs)

**Fig. 5:**
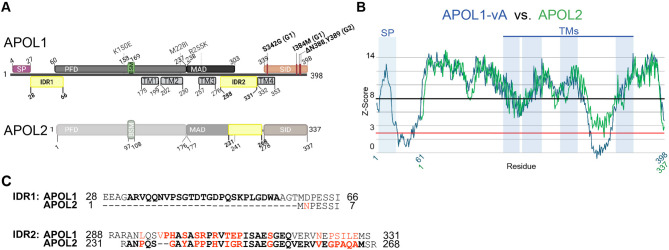
Determination of putative intrinsically disordered regions (IDRs) in APOL1. (**A**) Schematic representation of the two putative IDRs within the amino acid (aa) sequence of APOL1 (see Table 1 for details). (**B**) *In silico* analysis using the ADOPT prediction tool identified two IDRs in APOL1. For APOL2, one potential IDR was predicted, but with lower confidence (Z-score < 3). (**C**) Sequence alignment of the putative APOL1 IDR1 (aa 28–66) and IDR2 (aa 288–331) with the corresponding regions in APOL2. Bold letters indicate the IDR core sequence (IDR_min_ identified by at least two prediction tools); amino acids conserved between APOL1 and APOL2 are shown in black, differences in red.

The rapid degradation of the NT_vA_ chimera suggests that the N-terminal region of APOL1 may contain motifs that promote rapid protein degradation, and that this property can be transferred to APOL2. However, the rapid decline of APOL1 ΔN59 supports the existence of additional motifs promoting instability elsewhere in APOL1. Since proteasomal degradation requires protein unfolding, we next tested whether intrinsically disordered (and thus unfolded) regions (IDRs) can be identified within the APOL1 amino acid sequence. To address this, we applied five different *in silico* IDR prediction tools*(ADOPT, DisEMBL*,* flDPnn*,* IUPred2A*, and *AIUPred)*^28–32^. All tools identified two potential IDRs in APOL1: one in the N-terminus unique to APOL1 following the SP (core sequence IDR1_min_ aa 32–56), and another between transmembrane domains TM3 and TM4 (IDR2_min_ aa 297–317). Both IDRs are proximal to hydrophobic regions and Leucine zipper domains, while IDR1 directly follows the SP near to the PFD, and IDR2 is close to the MAD, two cholesterol recognition sites and the pore lining region^35–37^ (Fig. 5A). In contrast, only two of the five tools predicted an IDR in the orthologous region of APOL2 (IDR_min_ aa 232–268), and with markedly lower confidence (Table 1; Fig. 5A-C, Suppl. Fig. SF3). Together, the data indicate a potential correlation between the presence of IDRs and enhanced degradation of APOL1.

**Table 1:** Prediction of intrinsically disordered regions (IDRs).

Prediction software	Putative intrinsically disordered regions (IDRs)		
	APOL1 (vA)*		APOL2*
aa position IDR1	aa position IDR2	aa position IDR	
ADOPT	21–61	288–333	232–268
DisEMBL	26–66	293–341	231–270
flDPnn	30–60	294–317	–
IUPred2A	32–56	297–318	–
AIUPred	1–56	290–327	–
IDR_max_	IDR1_max_ 28–66	IDR2_max_ 288–331	IDR_max_ 231–270
IDR_min_	IDR1_min_ 32–56	IDR2_min_ 297–317	IDR_min_ 232–268

Using the IDR prediction tools ADOPT, DisEMBL, flDPnn, IUPred2A, and AIUPred the amino acid (aa) sequences of APOL1 (variant A) and APOL2 were analyzed for the presence of potential intrinsically disordered regions (IDRs). Only regions identified by at least two of the prediction tools, that consist of a region of more than 15 consecutive aa, and that were not already identified as putative transmembrane (TMs) spanning helixes (including the signal peptide, SP) were considered. IDR_max_ refers to the entire region predicted as a putative IDR by any of the selected tools; IDR_min_ indicates the core region that was identified as IDR by all five tools. The positions refer to the first and last beginning aa of the identified sequences.

*Amino acid sequences correspond to the UniProt identification numbers O14791 (for APOL1) and Q9BQE5 (for APOL2). The APOL1 predictions are also applicable to the African APOL1 haplotype.

### APOL1 surface pools are resistant to proteasomal degradation

As APOL1 degradation occurs independently of splice and risk variants (see Figs. 1, 2, 3 and 4), we next investigated whether intracellular localization affects APOL1 degradation. This is of particular interest, as minor amounts of APOL1 RRVs at the PM have been shown to drive cytotoxicity by causing an imbalance in the Na^+^/K^+^ ion homeostasis^9,16,35,38^.

To discriminate between total APOL1 expression levels (primarily resided in the ER) and the fraction of APOL1 at the cell surface, we used either the GFP fluorescence signal for total expression or an anti-APOL1 antibody able to detect APOL1 surface pools in unpermeabilized cells (Proteintech 11486-2-AP)^26,27^. Cells were treated with doxycycline (+ Dox) to induce expression, in combination with MG132 or CHX to inhibit degradation or biosynthesis processes, respectively.

**Fig. 6:**
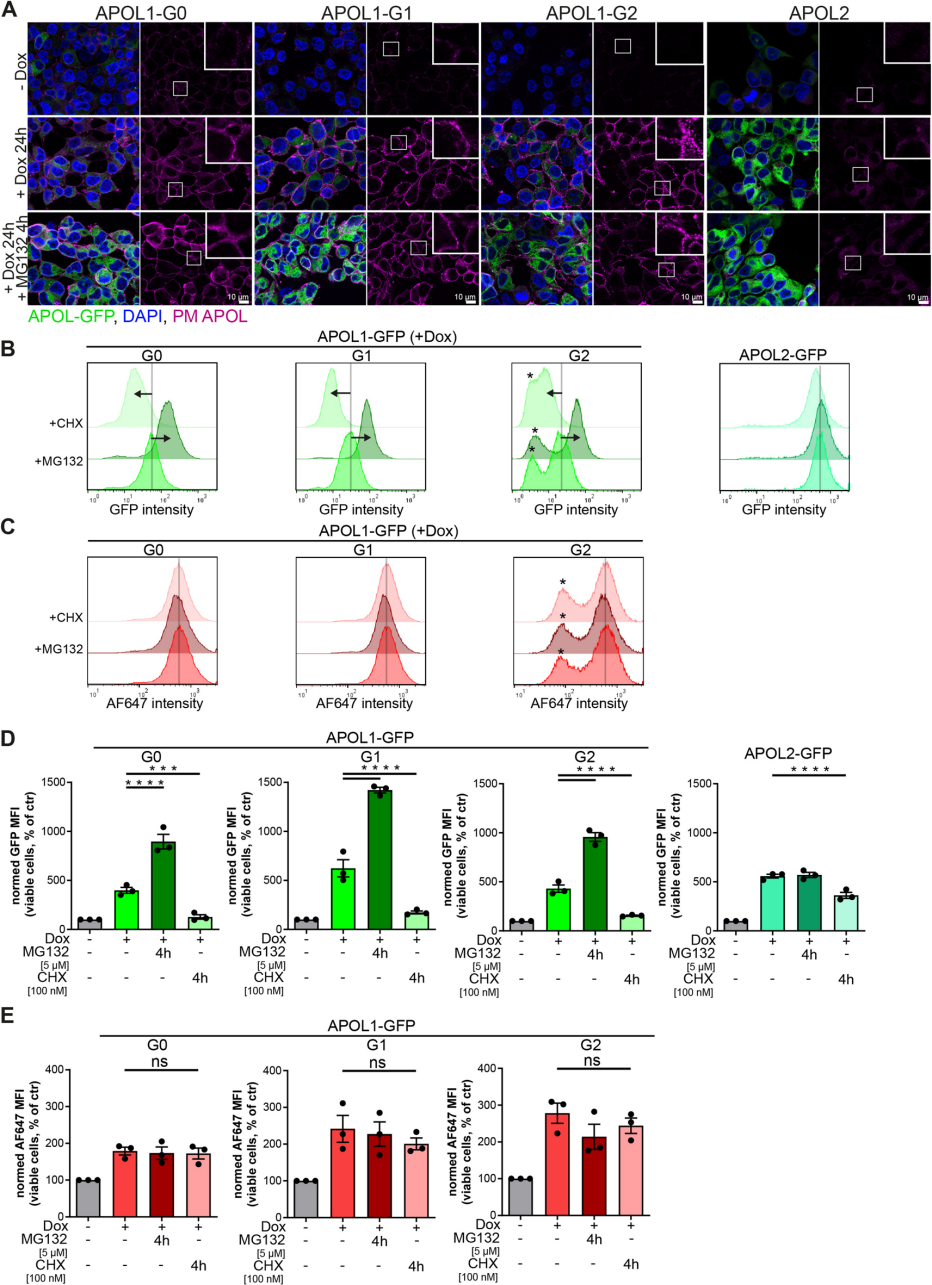
APOL1 surface pools are resilient against proteasomal degradation. (**A**) Immunofluorescence (IF) images of non-induced (-Dox), induced (+ Dox), and MG132-treated (+ Dox, +MG132) HEK293T cells expressing GFP-tagged APOL1 variants (G0 and RRVs) or APOL2. The GFP signal (green) indicates total cellular APOL1 expression. Cell surface-localized APOL1 was detected using an antibody recognizing extracellularly exposed epitopes (magenta). DNA counterstaining with DAPI. (**B**,**C**) Representative composite histograms showing total expression (**B**) and surface localization (**C**) of APOL1/APOL2 in HEK293T cells expressing GFP-tagged APOL1 G0, the RRVs G1 and G2, or APOL2. Conditions include non-induced cells (–Dox), induced cells (+ Dox), and induced cells additionally treated with MG132 or cycloheximide (CHX). (**D**,**E**) *Graphs*: FC analyses of cells summarized in B and C in combination with MG132 and CHX treatments shown as MFI values. Conditions include non-induced cells (–Dox), doxycycline-induced cells (+ Dox), and induced cells additionally treated with either MG132 or cycloheximide. (*N* ≥ 3). y-axis: cell count; x-axis: red fluorescence intensity (AF647) indicating surface-bound APOL1/APOL2. ns: not significant, ***: *p* < 0.001, ****: *p* < 0.0001.

The immunofluorescence (IF) studies show that expression levels of all untagged and GFP-tagged APOL1 (G0 and RRVs) and APOL2 were increased upon doxycycline administration, with a small dot-like fraction of APOL1 but not APOL2 at the cell surface (Fig. 6A, Suppl. Fig. SF4,SF5).

The same combination of cell lines and treatments used for IF analyses (+ Dox with MG132 or CHX) was also examined using FC analyses. As expected, GFP intensity – reflecting total APOL1 expression – increased upon MG132 treatment and decreased with CHX in APOL1 G0 and RRV variants. In contrast, APOL2 levels remained largely unchanged under these conditions. Surprisingly, fluorescence signals corresponding to APOL1 PM fractions (AF647) showed no detectable peak-shifts upon inhibition of proteasomal degradation or protein biosynthesis (Fig. 6B, C). Quantification of the mean fluorescence intensity (MFI), normalized to untreated controls (-Dox, -MG132, -CHX; Fig. 6D, E, Suppl. Fig. SF5), confirmed that PM-associated APOL1 levels were largely unaffected by MG132 or CHX treatment, indicating distinct degradation kinetics between intracellular and surface pools. While the total, predominantly intracellular APOL1 pools were sensitive to these treatments, the PM fractions - responsible for APOL1-linked cytotoxicity in the case of RRVs^9,39^ - remained highly resistant.

### APOL1 G0 and RRVs show similar surface expression levels at the cell surface

Comparing the intensity distribution of total expression levels and MFI values of GFP-tagged APOL1 and APOL2 expressing cell lines revealed distinct trends: the total (predominantly intracellular) pools are highest for APOL2 followed by non-toxic APOL1 and RRVs (G0 > G1 > G2). In contrast, PM-localized values of APOL1 showed relatively similar signal intensities (Fig. 6A, B). Together, this indicates that APOL1 PM pools are not only more resistant to proteasomal degradation but may also exhibit expression levels at the PM that are similar for all APOL1 variants (G0 and RRVs). Previous studies demonstrated direct correlation between APOL1-associated cytotoxicity and their expression levels. However, these studies have not distinguished between intracellular and cell surface fractions^20^.

**Fig. 7:**
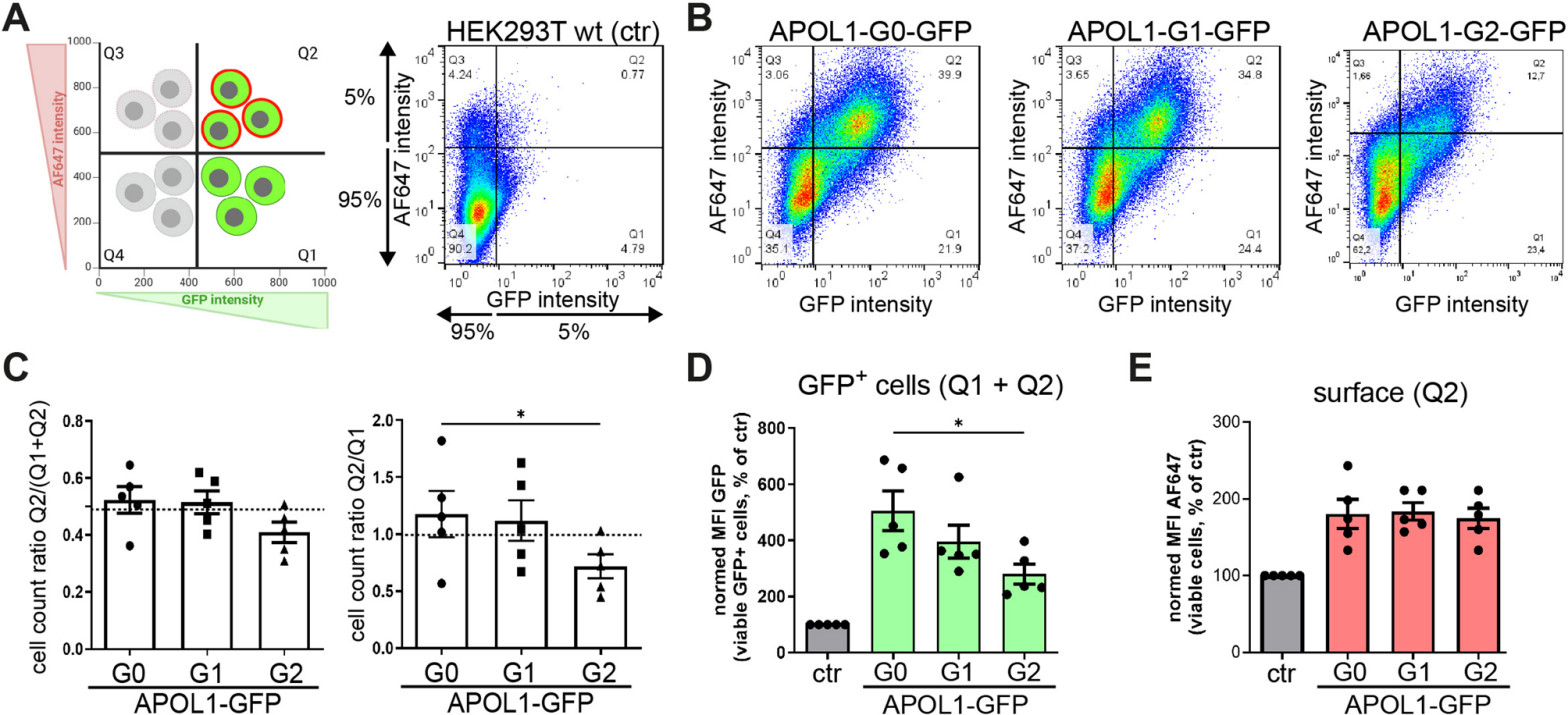
APOL1 surface and total expression after transient transfection in HEK293T cells. Transiently transfected HEK293T cells expressing GFP-tagged APOL1 variants (G0, G1, G2) or APOL2 (+ Dox 24 h). (**A**) Gating strategy. *Left*: definition of quadrants: Q1 (GFP^+^/AF647⁻), Q2 (GFP^+^/AF647^+^), Q3 (GFP⁻/AF647^+^, background only), and Q4 (GFP⁻/AF647⁻). *Right*: Thresholds were defined using the 95th percentile of background fluorescence of non-transfected HEK293T cells. (**B**) Scatter plots. Total APOL1-expressing cells are represented by Q1 + Q2 (GFP^+^ cells). Surface-localized APOL1 is captured by Q2 (GFP^+^/AF647^+^). (**C**) Quantification of surface expression across APOL1 G0 and RRVs. *Left*: Percentage of Q2 cells relative to total APOL1-expressing cells (Q1 + Q2). *Right*: Ratio of surface (Q2) to non-surface (Q1) APOL1-expressing cells. ns: not significant, *: *p* < 0.05. (**D**) Mean fluorescence intensities (MFIs) of GFP signal for APOL1-expressing cells (Q1 + Q2), indicating total expression levels. G0 shows the highest MFI, followed by G1 and G2. (**E**) MFI of AF647 fluorescence (Q2), indicating that surface pools of APOL1 levels are comparable across G0 and RRVs, despite differences in total expression.

Next, we simplified the approach by transiently transfecting HEK293T wildtype cells in doxycycline-containing medium, using identical plasmid concentrations. Thus, the only variable was the cDNA sequence encoding the different APOL1 variants (G0 or RRVs) or APOL2. After 24 h, APOL1 expression was analyzed by flow cytometry. We used non-transfected cells, which showed only low background signals for both red (AF647) and green (GFP) fluorescence in the FC analyses (Suppl. Fig. SF6A), to define the thresholds, set at the 95th percentile of the GFP and AF647 signals from these background measurements. For all subsequent analyses, only fluorescence intensities that exceeded these thresholds were considered. Based on these cut-off criteria, four quadrants (Q) were defined: Q1 contains cells that were positive for the green, but negative for the red fluorescence, reflecting cells in which APOL1 is significantly expressed, but not localized at the cell surface; Q2 presents cells in which APOL1 is expressed and also significantly found at the cell surface. Q3 encompasses signals that were negative for GFP fluorescence, but positive for AF647 signals. (As surface pools require total expression of APOL1-GFP these signals only reflect background signals), and finally Q4 that presents cells that were negative for both, the green and red fluorescence, thereby representing cells that do not express APOL1-GFP fusion proteins (Fig. 7A). Using these thresholds for evaluation, the representative scatter plots of transient transfection experiments elucidated expression of total APOL1 (Q1 + Q2 values) and that in all cases significant amounts of APOL1 are also localized at the cell surface (Q2 values, Fig. 7B, Suppl. Fig. SF6B).

Next, we quantified the fraction of cells with APOL1 surface expression relative to the total number of GFP-positive cells (Q1 + Q2, Fig. 7C, left graph), as well as the ratio of surface (Q2) to non-surface (Q1) APOL1 pools (Q2/Q1, Fig. 7C, right graph). Both analyses indicated that APOL1 G0 was expressed at higher overall levels than the RRVs, with G2 showing the lowest expression (Fig. 7C, Suppl. Fig. SF6C).

We also analyzed the MFI values of the different quadrants (Q1 and Q2), which not only reflect the number of cells above the defined thresholds but also provide information on fluorescence intensity across the cell population. The data summarized in Fig. 7D (Suppl. Fig. SF6B) show that the MFI of GFP-positive cells (Q1 + Q2) was highest for APOL1 G0 and lower for the RRVs (G1 > G2). However, when considering the MFI of the AF647 channel, which detects surface pools, all APOL1-expressing cells - G0 and both RRVs - showed similar values (Fig. 7E).

Together, these data indicate that although the RRVs express less total APOL1, surface expression levels are comparable to those of G0. These findings confirm the observations from the MFI analyses and suggest that APOL1 RRV surface pools that cause the cytotoxic effects are maintained despite lower total cellular expression.

## Discussion

It has been shown in various *in vitro* and *in vivo* systems that APOL1-mediated cytotoxicity depends on two essential key factors: the (homozygous) presence of APOL1 RRVs, in an African genetic background^39^, and second, a certain APOL1 expression level (or threshold), which is able to trigger cytotoxic effects^20^. Protein expression levels are not only determined by increased transcription and subsequent translation of the gene but also by the protein degradation rate. Of note, high protein stability, low protein turnover or reduced protein degradation can lead to elevated endogenous protein levels even after induced transcription has ceased. This is of particular importance, as the inflammatory and immunomodulatory signals that regulate *APOL1* gene expression can be of both transient (temporary) and chronic nature^3,4^.

However, in this study we unexpectedly observed that APOL1 is rapidly degraded via the proteasome, suggesting that the APOL1 cytotoxicity cannot be simply explained by its persistence of APOL1 due to low degradation rates. Moreover, similar degradation rates of the wildtype G0 and the RRVs (G1, G2) of APOL1 were observed. This suggests that APOL1 cytotoxicity is unlikely to arise from differential stability or degradation of APOL1 G0 and RRVs leading to locally elevated RRV levels.

Furthermore, our studies show that not only the main variant A of APOL1, but also all other isoforms are degraded much faster than APOL2, which is similar in sequence and evolutionarily most closely related to APOL1^22,40^. Even the APOL1 variant vB3, which lacks the SP and therefore exhibits a similar cytoplasmic orientation or membrane topology to APOL2, undergoes rapid degradation^22,26^. Thus APOL1’s rapid degradation does not depend on RRVs, nor does it depend on an ER-lumen or cytoplasmic faced membrane orientation of its N- and C- terminal ends.

The subsequent search for sequence motifs that might explain the differences in degradation dynamics between APOL1 and APOL2 (such as PEST sequences) led to the identification of two intrinsically disordered regions – IDR1, aa 28–66 and IDR2, aa 288–331 – which are present in APOL1 but, interestingly, absent from the APOL2 sequence. IDRs are regions within a protein that lack a stable 3D structure and are therefore unstructured or partially unfolded. These sequence motifs typically span around 30 aa or more and are found in many human transmembrane proteins, especially on the cytoplasmic side^41,42^.

In addition to their involvement in biological functions such as ion channel regulation and signal transduction, increasing evidence suggests that IDRs also regulate protein degradation. Due to their structural flexibility, IDRs often mediate rapid proteasomal degradation, particularly in a ubiquitin-independent manner^43,44^, thereby markedly shortening protein half-life^45,46^. These processes may predominantly involve the 20S proteasome, as - in contrast to the 26S proteasome, which mainly degrades ubiquitinated proteins - the 20S proteasome preferentially degrades unfolded or misfolded proteins as well as intrinsically disordered proteins (IDPs) or proteins containing IDRs^47,48^. The proteasome inhibitors used in this study (MG132 and Bortezomib) do not allow a clear distinction between these degradation pathways. Thus, it remains unclear whether APOL1 is mainly degraded by the 20S or the 26S proteasome, and whether the different APOL1 isoforms are degraded in different ways. Moreover, the question of whether APOL1 undergoes classical ubiquitination has not been thoroughly investigated. Previous studies showed that the ubiquitin-like protein FAT10 (also called ubiquitin D, UBD) binds APOL1 and that elevated UBD levels can decrease cytotoxicity linked to APOL1 RRVs^49^. However, to what extent the identified IDR1 and IDR2 are involved in the APOL1-FAT10 interaction needs to be clarified in further studies.

Of note, transferring the N-terminal region of APOL1, which includes the IDR1 motif, to the N-terminus of APOL2 accelerated the degradation of the NT_vA_-APOL2 fusion protein, which is also oriented towards the ER lumen. This suggests that IDR1, present in nearly all APOL1 splice isoforms, may contain a core region that promotes NT_vA_-APOL2 degradation. Whether the degradation mediated by IDR1 in APOL1 and NT_vA_-APOL2 primarily involves ER-associated degradation (ERAD), with retro-translocation of proteins from the ER into the cytosol, remains to be determined. In this context it is interesting that ER-translocon-dependent processes have been shown to be important not only for APOL1 ER insertion but also for APOL1-associated cytotoxicity^14^. However, the observation that APOL1 deletion mutant ΔN59, which lacks IDR1, is also rapidly degraded, indicates that the IDR1 is not the only determinant of rapid degradation of APOL1.

The second IDR of APOL1 (IDR2_max_ aa 288–331) is located between the transmembrane domains TM3 and TM4. Parts of this region have undergone positive selection during evolution^22,40^ and has previously been associated with various conformational changes^27,35,50^. Notably, *Gupta et al.* observed that region within APOL1 (aa 235–314, which shares 26 residues with IDR2) that was recognized by certain anti-APOL1 antibodies in non-human CHO cells, but not on the surface of human immortalized podocytes, despite both cell types expressing the same APOL1 protein variant^26,27^. The authors suggested that this discrepancy might be due to protein-protein or protein-lipid interactions that mask the recognition of epitopes in this region in human cells. However, another possibility might be that APOL1 is expressed in different conformations on the cell surface, including the possibility that there are APOL1 pools with two, two and a half-membrane spanning loop, or four transmembrane helices^18,22,27^.

Evaluation of the *surface-to-total* expression rations (based on the FC data) revealed an interesting trend showing that APOL1 RRVs require less total expression levels to reach similar surface levels as the wildtype. In this context it is interesting that similar FC studies from *Gupta et al.* showed that the RRVs are faster exported from the ER via the secretory pathways to the surface than the APOL1 wildtype^39^.

Our observations suggest that the fraction of APOL1 present at the cell surface is either more stably folded than the ER-associated pools, has evaded proteasomal degradation during transport through the secretory pathway, or both. Thus, our data highlight two key aspects: first, they indicate that, following expression induced by immunomodulatory triggers, the balance between APOL1 processing (translation and folding), degradation, and transport determines its surface expression. Second, the resistance of membrane-associated APOL1 pools to degradation may be a critical factor that increases or stabilizes their presence at the cell surface. These pools appear particularly relevant for the cytotoxic effects of APOL1 RRVs, as they function as pores at the PM^9,16,35,38^, in contrast to APOL1 G0 (wildtype), disrupting ion homeostasis and triggering downstream cellular damage.

## Conclusions

Our study is based on a simplified *in vitro* system (HEK293T cells) using overexpression of GFP-tagged APOL1 fusion proteins and we cannot exclude the existence of further tissue- and cell-type-specific APOL1 isoforms. It also remains unclear whether different isoforms are co-expressed within the same cells and whether they influence each other. Overall, our findings - consistent with those from numerous other groups - indicate that obvious cell biological differences between non-toxic APOL1 wildtype (G0) and RRVs are difficult to detect.

All variants can be induced by inflammatory triggers, and both APOL1 G0 and RRVs show similar intracellular localizations, comparable degradation dynamics, and similar levels at the cell surface. Thus, APOL1-associated cellular injury (or APOL1 linked pathogenicity) most likely arises from dynamic processes at the ER, such as the balance between misfolded and correctly folded proteins, the relative abundance of different conformations within cellular compartments, or the kinetics and stability of APOL1 complexes within intracellular pools or at the cell surface. The presence of IDRs emphasizes APOL1’s high structural flexibility. Notably, such dynamic features may vary substantially between cell types, potentially explaining why certain human cells* in vivo *– such as podocytes – are more susceptible to APOL1-associated damage than others. These aspects warrant further investigation into future studies.

## Supplementary Information

Below is the link to the electronic supplementary material.

Supplementary Material 1

Supplementary Material 2

## Data Availability

The materials used during the current study are available from the corresponding author on reasonable request.

## Abbreviations

AF647: AlexaFluor™ 647
AMKDs: APOL1-mediated kidney diseases
BSA: bovine serum albumin
CHX: Cycloheximide
COVAN: Covid-19 associated nephropathy
DAPI: 4′,6-diamidino-2-phenylindole
DMEM: Dulbecco’s modified eagle’s medium
DMSO: Dimethyl sulfoxide
Dox: doxycycline
ECL: Enhanced chemiluminescence
ER: endoplasmic reticulum
ERAD: ER-associated degradation
FC: flow cytometry
FSC: forward scatter
GFP: green fluorescent protein
H: hour
HEK: Human embryonic kidney
HIVAN: HIV-associated nephropathy
IDRs: Intrinsically disordered regions
IF: Immunofluorescence
MFI: Mean fluorescence intensity
Min: Minute
PBS: Phosphate-buffered saline
PFA: Paraformaldehyde
PNGase F: Peptide: N-glycosidase F
RPMI: Roswell Park Memorial Institute
RRVs: Renal risk variants
SDS PAGE: sodium dodecyl sulfate–polyacrylamide gel electrophoresis
SSC: Sideward scatter
TBST: Tris-buffered saline with Tween 20
TM: Transmembrane domain
UBD: ubiquitin D/FAT10
vA, vB1, vB3, vC: Variant/isoforms A, B1, B3,C
WB: Western blot
