# Supplementary material for: APOL1 plasma membrane pools resist rapid protein degradation
Source: Sci Rep. 2026 Feb 16;16:6718. doi: 10.1038/s41598-026-37647-z (PMC12913894; doi:10.1038/s41598-026-37647-z)
Supplement: Supplementary file 1 — Supplementary Material 1 [file 41598_2026_37647_MOESM1_ESM.pdf]

Supplemental Material

APOL1 plasma membrane pools resist rapid protein degradation.

by

Verena Höffken[1], Laura Alvermann[1], David Niggemeier[1], Katrin Beul[1], Pavel Nedvetsky[1], Bernhard Ellinger[2], Daria Assenmacher[1], Daniel Granado[1], Hermann Pavenstädt[1], and Thomas Weide[1*]

[1)] University Hospital Münster, Medical Clinic D, Albert Schweitzer-Campus 1, Geb. A14, 48149 Münster, Germany

[2)] Fraunhofer Institute for Translational Medicine and Pharmacology ITMP, Department Screening Port, Schnackenburgallee 114, 22525 Hamburg, Germany

Correspondence: Thomas Weide: weidet@uni-muenster.de

Content:

Suppl. Fig. SF1: *APOL1 is degraded via the proteasome.*

Suppl. Fig. SF2: *APOL1 and APOL2 show different degradation dynamics.*

Suppl. Fig. SF3: *Determination of putative intrinsically disordered regions (IDRs) in APOL1*.

Suppl. Fig. SF4: *APOL1 surface pools in untagged cells.*

Suppl. Fig. SF5: *APOL1 surface pools are resilient against proteasomal degradation*.

Suppl. Fig. SF6: *APOL1 surface and total expression after transient transfection in HEK293T cells*.

Table ST1: *Used primers for cloning cDNA encoding the* NT_{vA}*-APOL2 protein.*

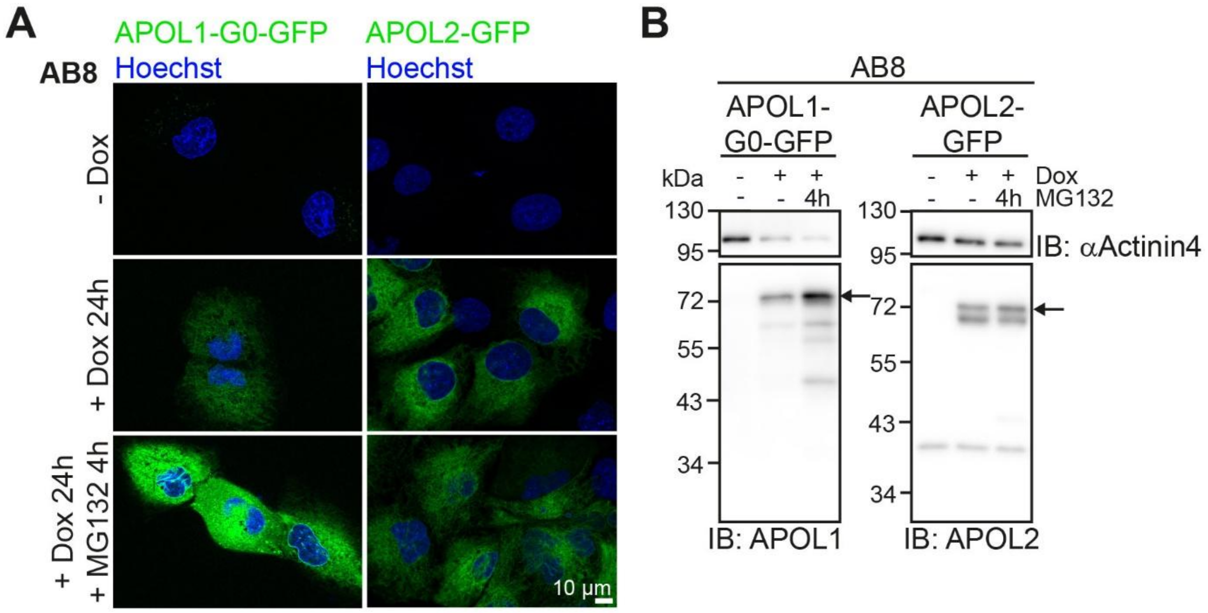

Suppl. Fig. SF1: *APOL1 is degraded via the proteasome.*

Immortalized human podocytes (AB8 cells) enabling a doxycycline-dependent expression of C-terminally GFP-tagged APOL1 wildtype (G0) and APOL2 were treated with proteasome inhibitors MG132. **(A)** Live cell images of AB8 cells expressing APOL1 G0 or APOL2. *Upper panel:* non-induced cells (-Dox). *Middle panel:* APOL1 and APOL2 cells in which expressions were triggered with 125 ng/ml doxycycline for 24 h (+Dox). *Lower panel:* Dox-induced cells with an additional MG132 treatment (5 µM) for 24 h (+Dox, +MG132). **(B)** Western Blot analysis of cells shown in A using specific antibodies against APOL1 and APOL2. α-Actinin4 served as loading control.

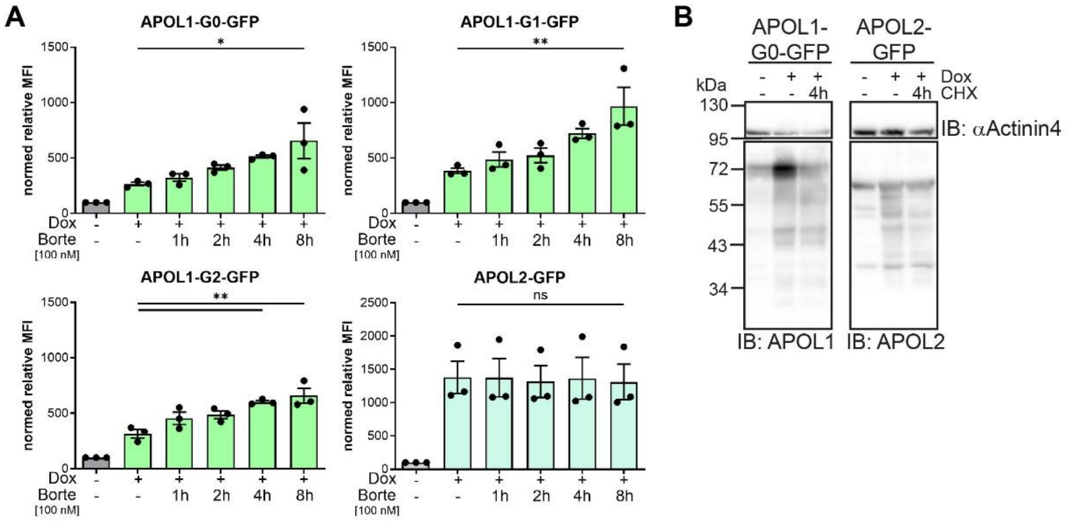

Suppl. Fig. SF2: *APOL1 and APOL2 show different degradation dynamics.*

HEK293T cell lines enabling a doxycycline-dependent expression of C-terminally GFP-tagged APOL1 wildtype (G0) and APOL2 were treated with proteasome inhibitor drug Bortezomib. **(A)** *Graphs:* FC analyses of non-induced, induced APOL1 (G0, and RRVs in green) and APOL2 (turquoise) cells with or without Bortezomib treatment (100 nM) for different periods (1 h, 2 h, 4 h, or 8 h) summarized using the mean fluorescence intensity (MFI). The analyses show FC analyses of at least three independent experiments (N ≥ 3). **(B)** Western Blot analysis of non-induced, induced and CHX treated HEK293T cells expressing APOL1 G0 and APOL2 using specific antibodies against APOL1 and APOL2. α-Actinin4 served as loading control. ns: not significant, *: $p < 0.05$, **: $p < 0.01$.

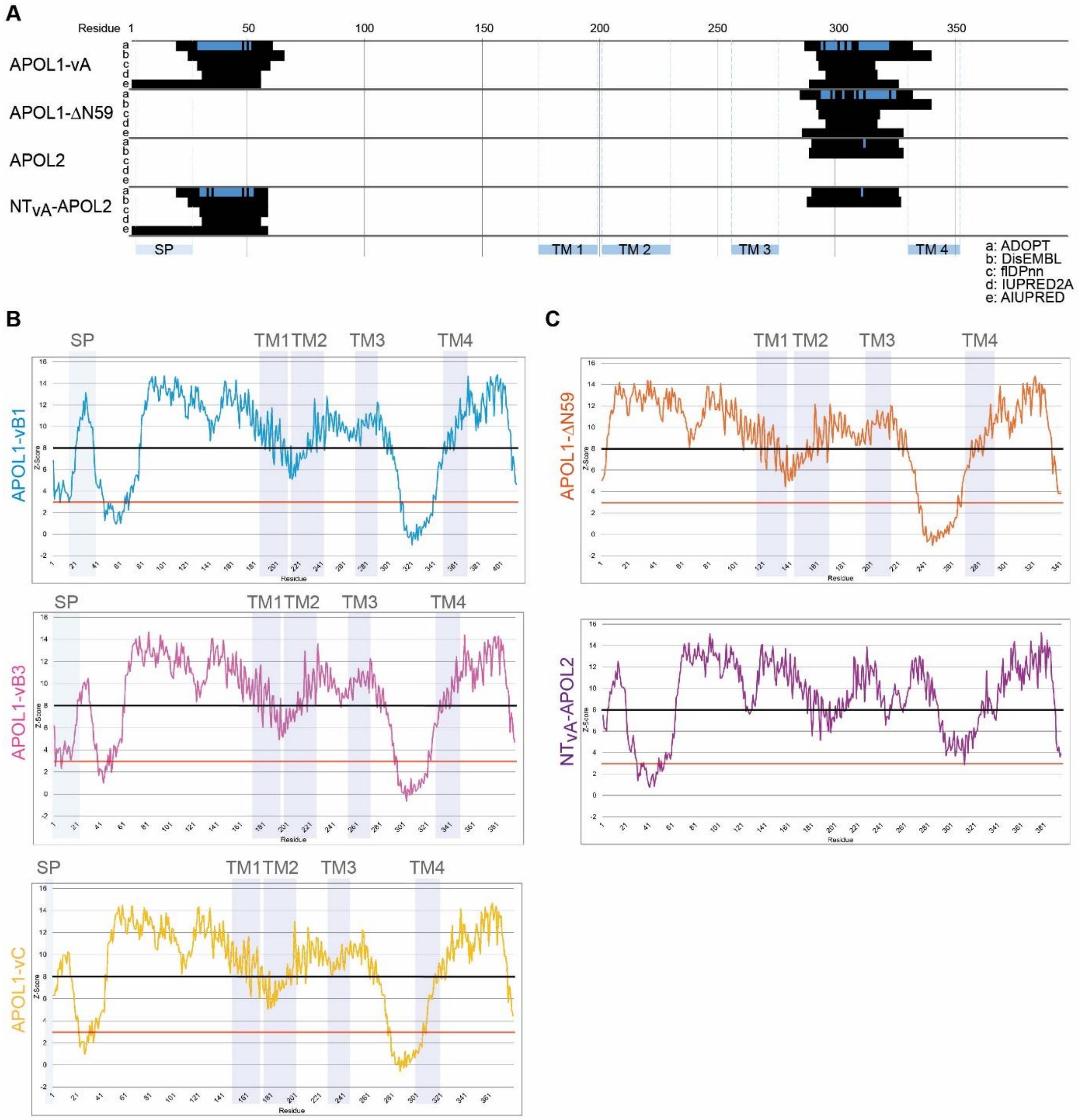

Suppl. Fig. SF3: *Determination of putative intrinsically disordered regions (IDRs) in APOL1*.

(A) Schematic representation of intrinsically disordered regions (IDRs) in APOL1, ΔN59-APOL1, APOL2, and NT_{vA}-APOL2, as predicted by the tools ADOPT, DisEMBL, flDPnn, IUPred2A, and AIUPred. Predicted IDRs are indicated by black boxes (blue boxes indicate stronger ADOPT threshold, Z score < 3). Putative transmembrane (TM) helices or membrane-interacting regions are shown in bluish and labeled as TM. **(B, C)** Graphical representation of the *in silico* analysis results from the ADOPT prediction tool for APOL1 splice variants vB1, vB3, and vC, the deletion mutant ΔN59-APOL1, and the chimeric fusion protein NT_{vA}-APOL2. Intrinsically disordered regions (IDRs) are indicated by regions with elevated Z-scores (black and red lines).

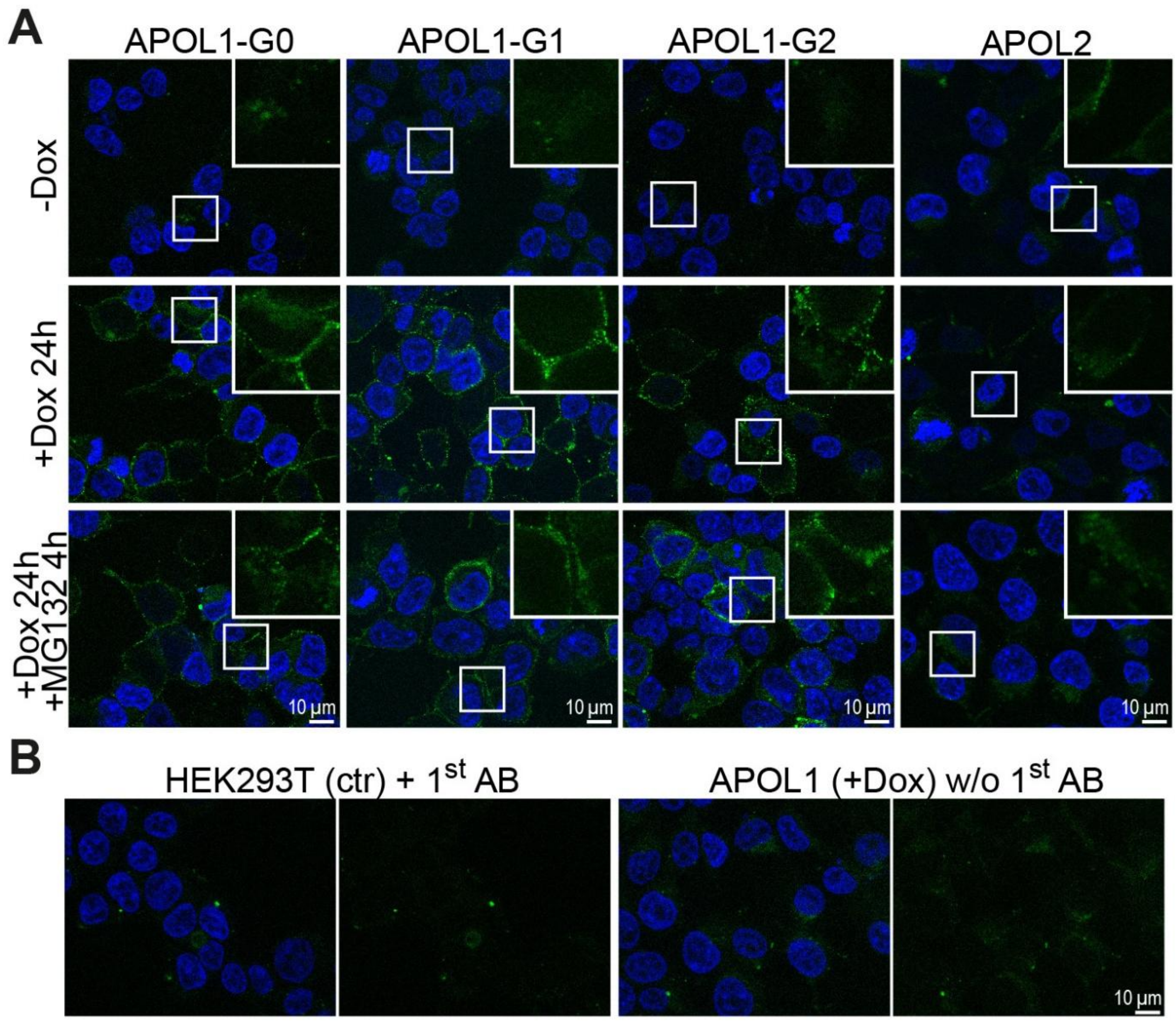

Suppl. Fig. SF4: *APOL1 surface pools in untagged cells.*

(A) Immunofluorescence (IF) images of non-induced (-Dox), induced (+Dox), and MG132-treated (+Dox, +MG132) HEK293T cells expressing untagged APOL1-G0 and RRVs or APOL2. Cell surface-localized APOL1 was detected using an antibody recognizing extracellularly exposed epitopes labelled with AlexaFluor®488 (Invitrogen, A11034; green). DNA counterstaining with DAPI. **(B)** Immunofluorescence (IF) images of staining controls: HEK293T cells stained against APOL1 on the surface and 24 h Dox-induced untagged APOL1 cells stained without the primary antibody (omission control). Cell surface-localized APOL1 (green) was not present in both controls. DAPI served as counterstaining.

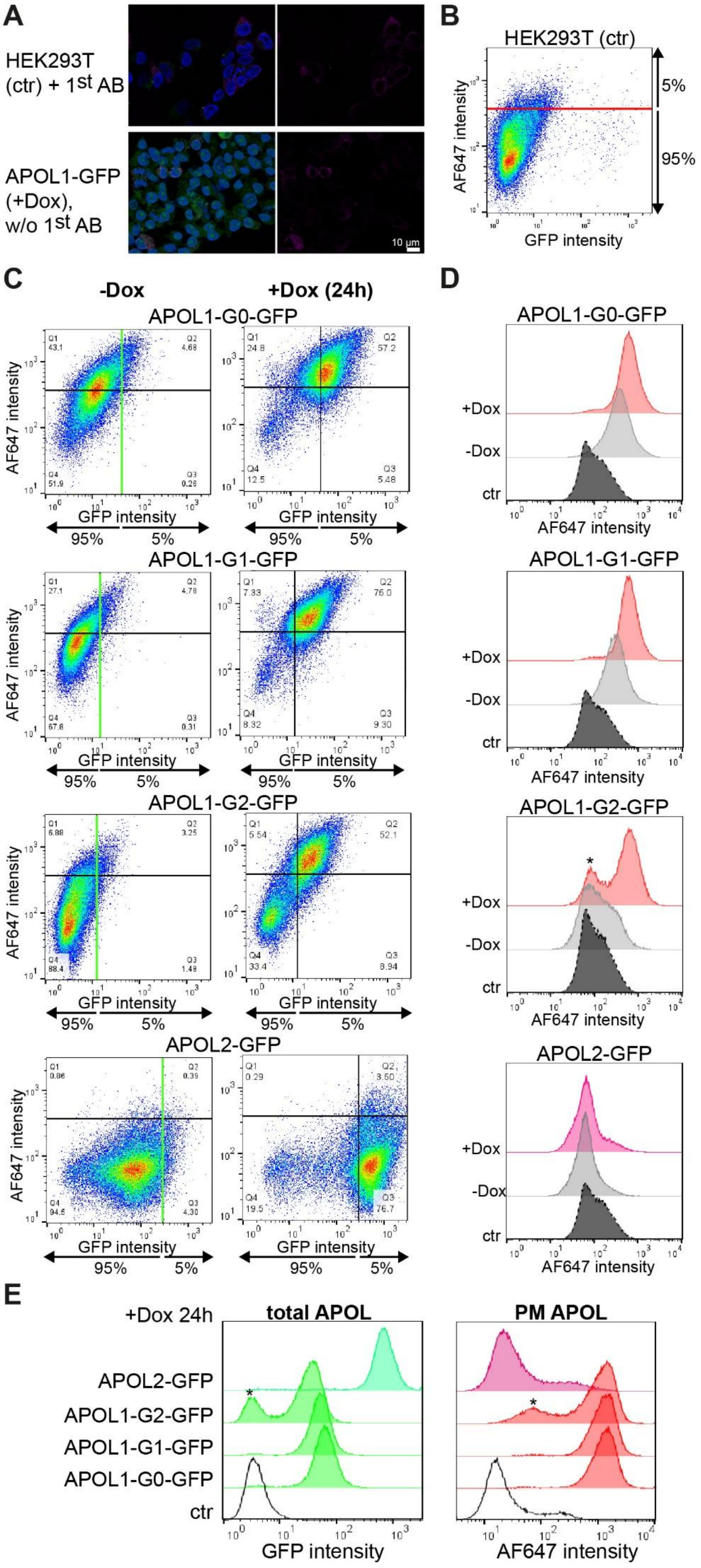
A
HEK293T
(ctr) + 1st AB
APOL1-GFP
(+Dox),
w/o 1st AB
10 µm
B
HEK293T (ctr)
AF647 intensity
GFP intensity
5%
95%
C
-Dox
+Dox (24h)
APOL1-G0-GFP
APOL1-G1-GFP
APOL1-G2-GFP
APOL2-GFP
AF647 intensity
GFP intensity
95%
5%
D
APOL1-G0-GFP
APOL1-G1-GFP
APOL1-G2-GFP
APOL2-GFP
+Dox
-Dox
ctr
AF647 intensity
E
+Dox 24h
total APOL
PM APOL
APOL2-GFP
APOL1-G2-GFP
APOL1-G1-GFP
APOL1-G0-GFP
ctr
GFP intensity
AF647 intensity

Suppl. Fig. SF5: *APOL1 surface pools are resilient against proteasomal degradation*.

(A) Immunofluorescence (IF) images of staining controls: HEK293T cells stained against APOL1 on the surface and 24 h induced GFP-tagged APOL1 cells stained without the primary antibody. While green indicates autofluorescence of wt or total cellular APOL1 expression, cell surface-localized APOL1 (magenta) was not present in both controls. DAPI served as counterstaining. **(B)** Representative scatter plot of HEK293T control cells used for gating flow cytometry data. The 95th percentile of AF647 intensity in control cells stained against surface APOL1 was used to define positive APOL1 surface staining. **(C)** Representative scatter plots of GFP and AF647 signals of stable APOL-GFP cell lines. The gate for GFP positive cells was set in uninduced (-Dox) conditions per stable cell line based on the 95th percentile and applied to the induced (+Dox) conditions. In combination with the gate for positive surface signal (B), quartiles Q1-Q4 are defined. Induction of cell lines lead to increased GFP intensity and for APOL1 cell lines also increased AF647 signal. For B, C: x-axis: GFP fluorescence; y-axis: AF647 fluorescence. **(D)** Representative histograms of AF647 intensity of APOL cell lines and ctr. While for APOL1 cell lines an increased intensity of surface APOL signal is visible after induction (+Dox, 24 h), APOL2 intensities remain similar to the ctr. Asterisk indicates uninducible subpopulation in APOL1-G2-GFP cell line. **(E)** Representative overlayed histograms of GFP and AF647 signals of APOL-GFP cell lines and ctr cells. Asterisk indicates uninducible subpopulation in APOL1-G2-GFP cell line. Histograms: x-axis: GFP/AF647 fluorescence; y-axis: cell count.

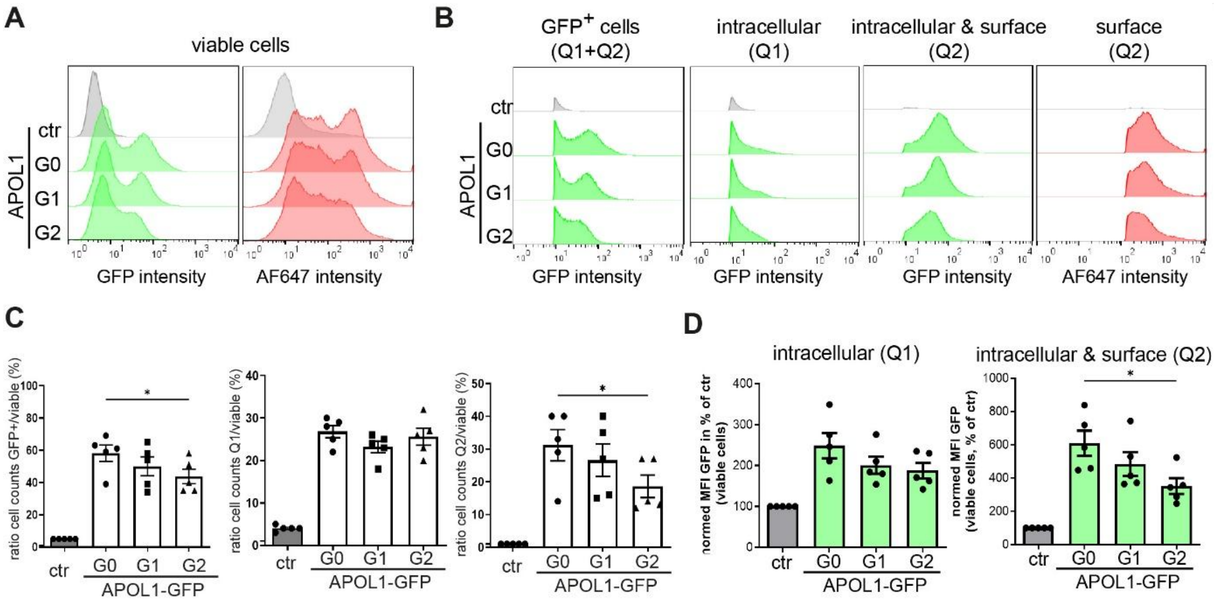

Suppl. Fig. SF6: *APOL1 surface and total expression after transient transfection in HEK293T cells*. **(A)** Representative overlayed histograms of GFP and AF647 signals of viable ctr and HEK cells transfected with APOL1-GFP constructs for G0 and risk variants G1 and G2. **(B)** Representative overlayed histograms of fluorescence intensities of viable ctr and transiently transfected APOL1-GFP cells. GFP^{+} cells display the positively transfected and induced cell populations (quartiles Q1 and Q2). GFP intensity in cells with only intracellular APOL1 are displayed by quartile Q1, cells with intracellular and surface APOL1 by Q2. AF647 intensity represents the APOL1 surface signal in cells with intracellular and surface APOL1. Gating was performed as described on ctr cells. **(C)** Ratio of cell counts of GFP^{+} cells to all viable cells for ctr and APOL1-GFP transfected cells show increased ratio indicating transfection rate in APOL1-G0-GFP transfected cells compared to ctr but decreasing ratios in G1 and significantly in G2 transfected ones. Ratios differed not significantly between APOL1 transfections in the quartile Q1 to viable cell ratio, but again in quartile Q2 to viable cell ratio showed decreasing trend with significance to G2. **(D)** Mean fluorescence intensity (MFI) of GFP signal of viable cells normed on ctr. GFP MFI in cells with intracellular APOL1 (Q1) showed a not significant decreasing trend correlating with risk variants. In cells with intracellular and surface APOL1 (Q2) the GFP MFI shows a similar decreasing trend, significantly reduced in G2 compared to G0. ns: not significant, *: $p < 0.05$.

SUPPLEMENTAL TABELS

Table ST1: *Used primers for cloning cDNA encoding the NT_{vA}-APOL2 protein.*

Name	Sequence 5‘-3‘
Amplification of the NTvA and APOL2 N-term cDNA	
fwd1	caccgctagcagatctatggagggagctgctttgctgagagtctctgtcctctgcatctggatgagtgcacttttccttggtgtgggagtgaggg
rev1	aaaagtgcactcatccagatgcagaggacagagactctcagcaaagcagctccctccatctcgagatctgagtccggta
fwd2	ccttggtgtgggagtgagggcagaggaagctggagcgagggtgcaacaaaacgttccaagtgggacagatactggagat
rev2	ttggaacgttttgttgcaccctcgctccagcttcctctgccctcactcccacaccaaggaaaagtgcactcatccagat
fwd3	gtgggacagatactggagatcctcaaagtaagcccctcggtgactgggctgctggcaccaacccagagagcagtatctt
rev3	ggtgccagcagcccagtcaccgaggggcttactttgaggatctccagtatctgtcccacttggaacgttttgttgcacc
Adding the cDNA encoding the Glyco-tag	
for 4	atggagggagctgctttgc
rev4	cttaattaactagatgtcccaaacgtcaccagaagcggccgcgacggtagcgttagcagcagcagcagcagccttgtacagctcgtccatgccgag
