# Supplementary material for: APOL1 plasma membrane pools resist rapid protein degradation
Source: Sci Rep. 2026 Feb 16;16:6718. doi: 10.1038/s41598-026-37647-z (PMC12913894; doi:10.1038/s41598-026-37647-z)
Supplement: Supplementary file 2 — Supplementary Material 2 [file 41598_2026_37647_MOESM2_ESM.pdf]

Uncropped Western Blot images

Figure 1A:

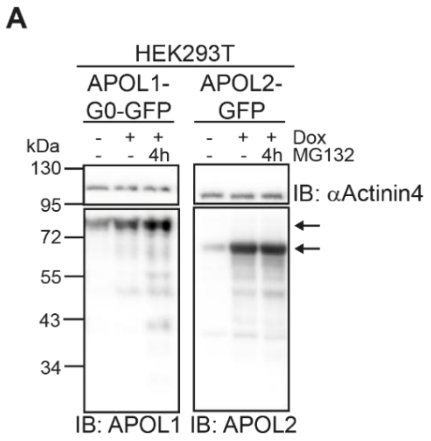

Fig. 1: *APOL1 is degraded via the proteasome.*

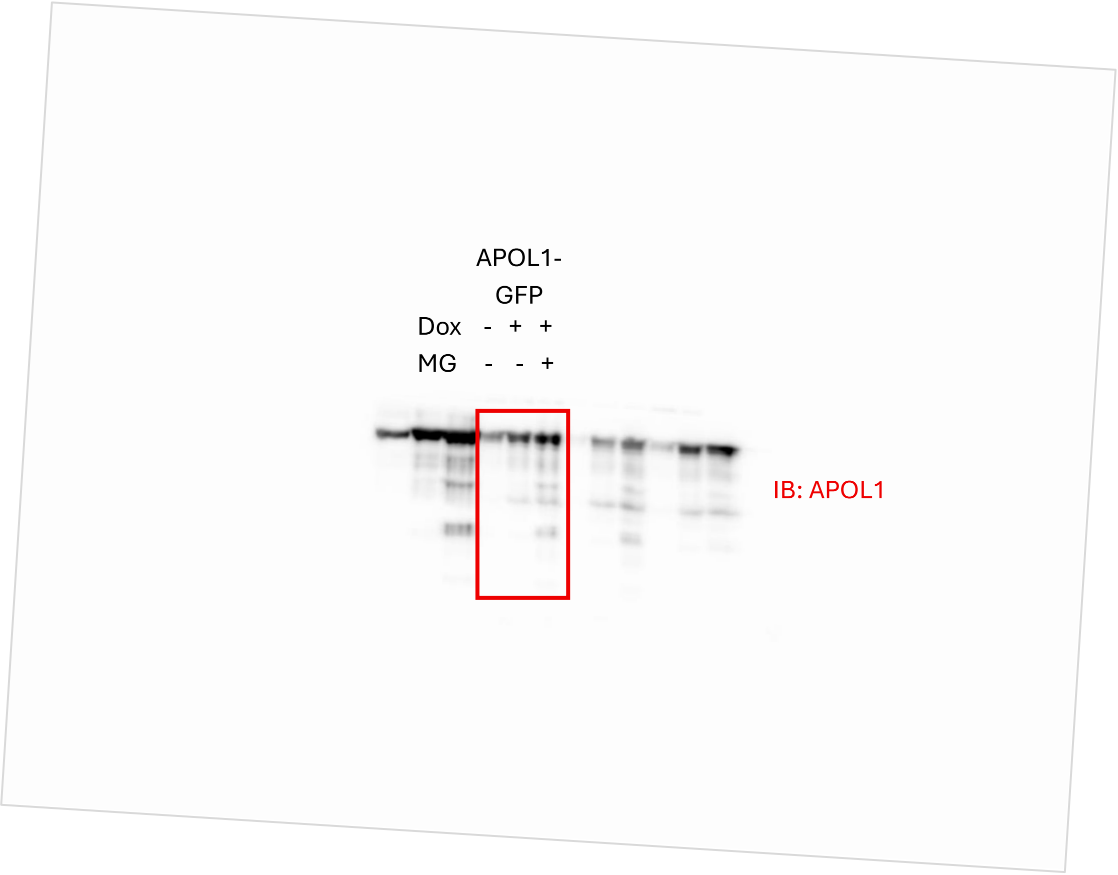

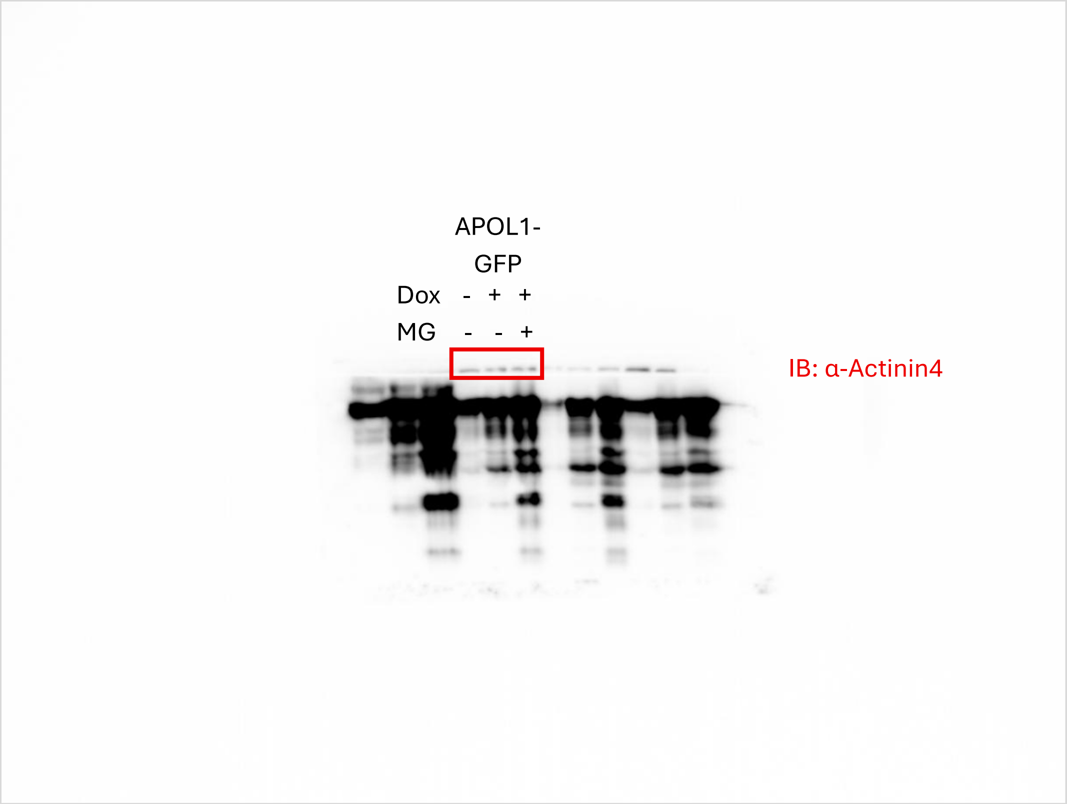

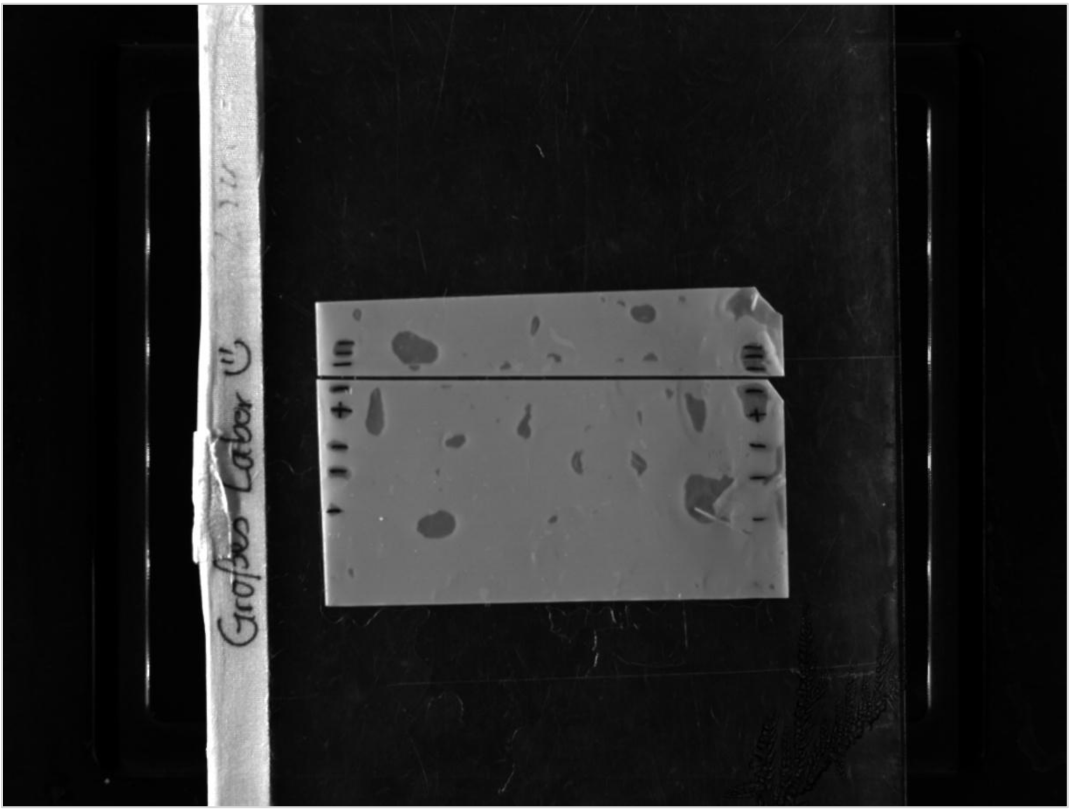

corresponding marker image

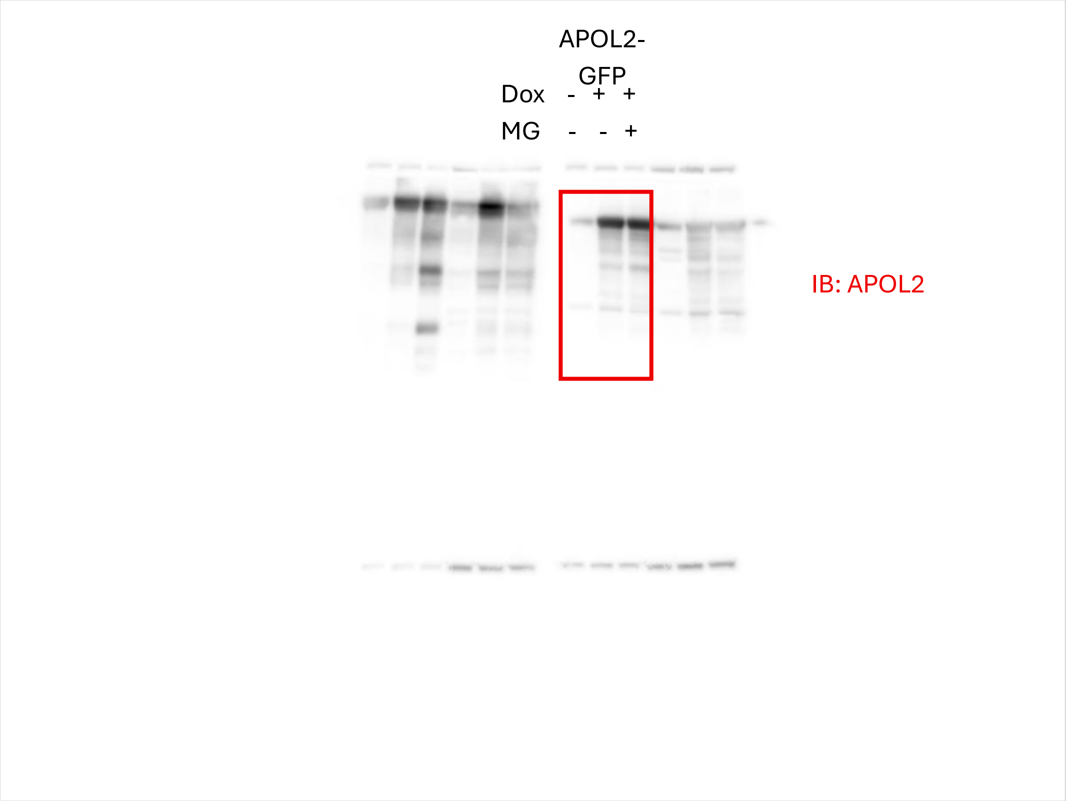
APOL2-GFP
Dox - + +
MG - - +
IB: APOL2

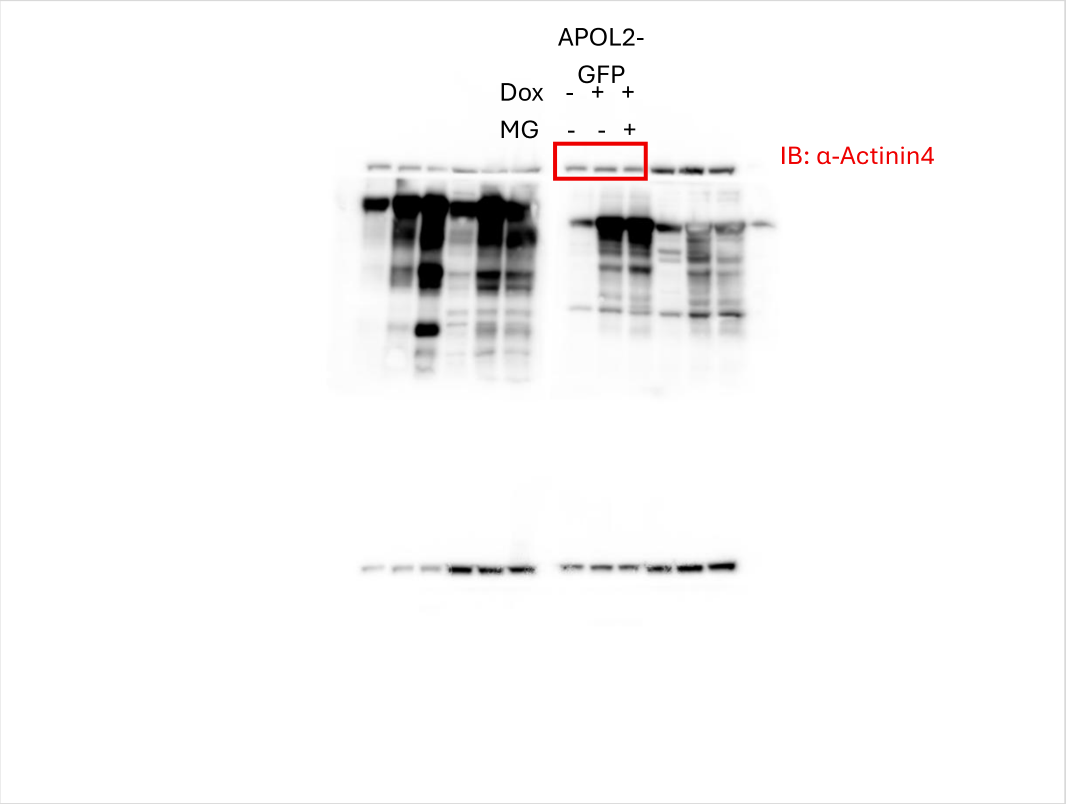
APOL2-GFP
Dox - + +
MG - - +
IB: α-Actinin4

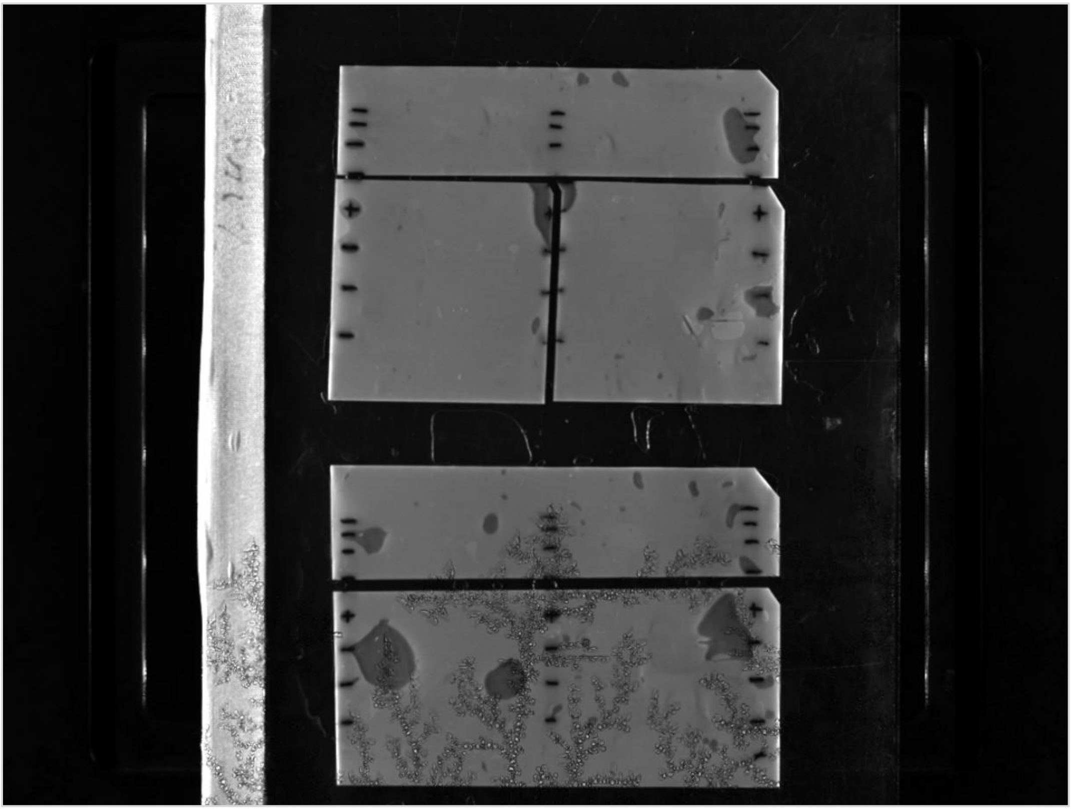

corresponding marker image

Suppl. Figure SF1 B:

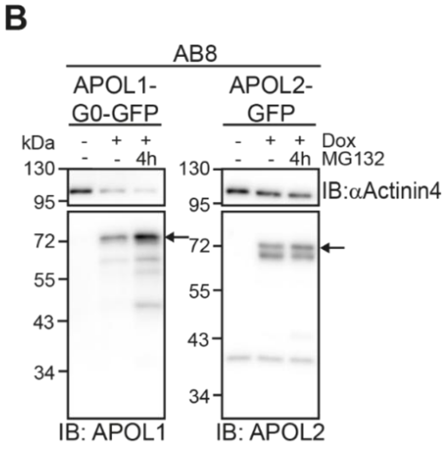

Suppl. Fig. SF1: *APOL1 is degraded via the proteasome.*

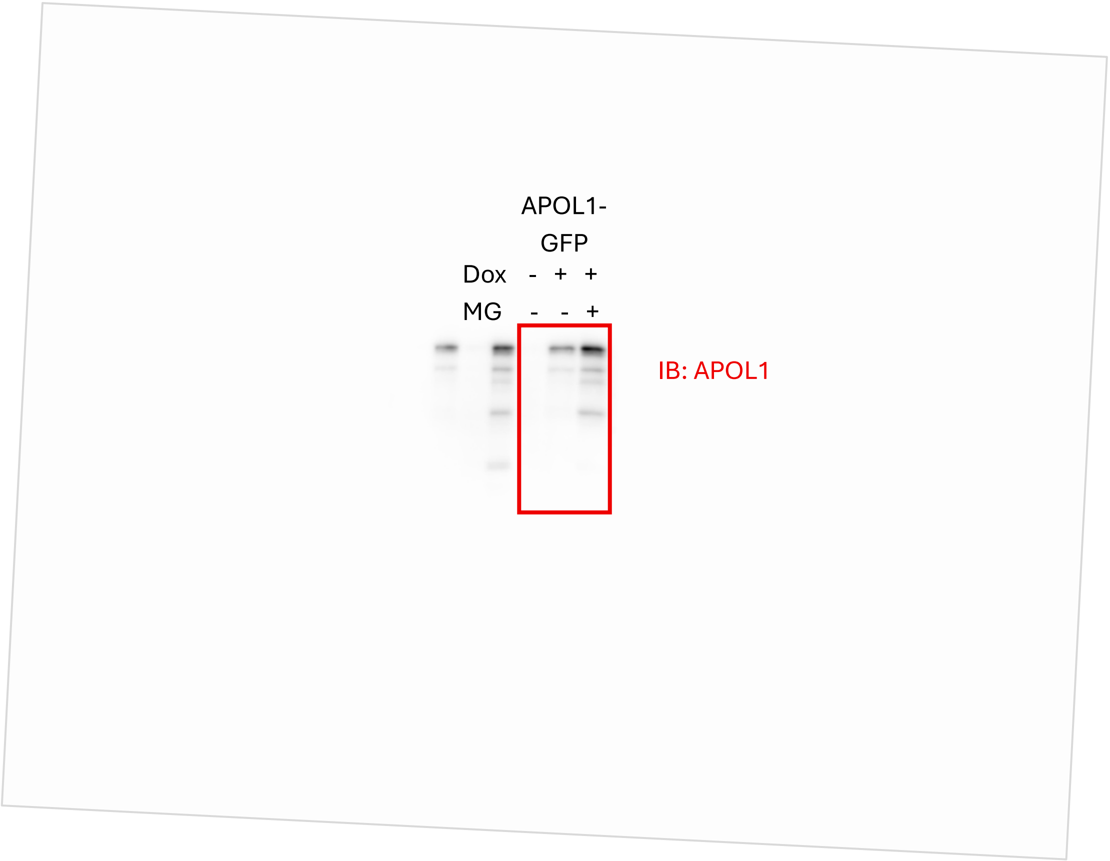

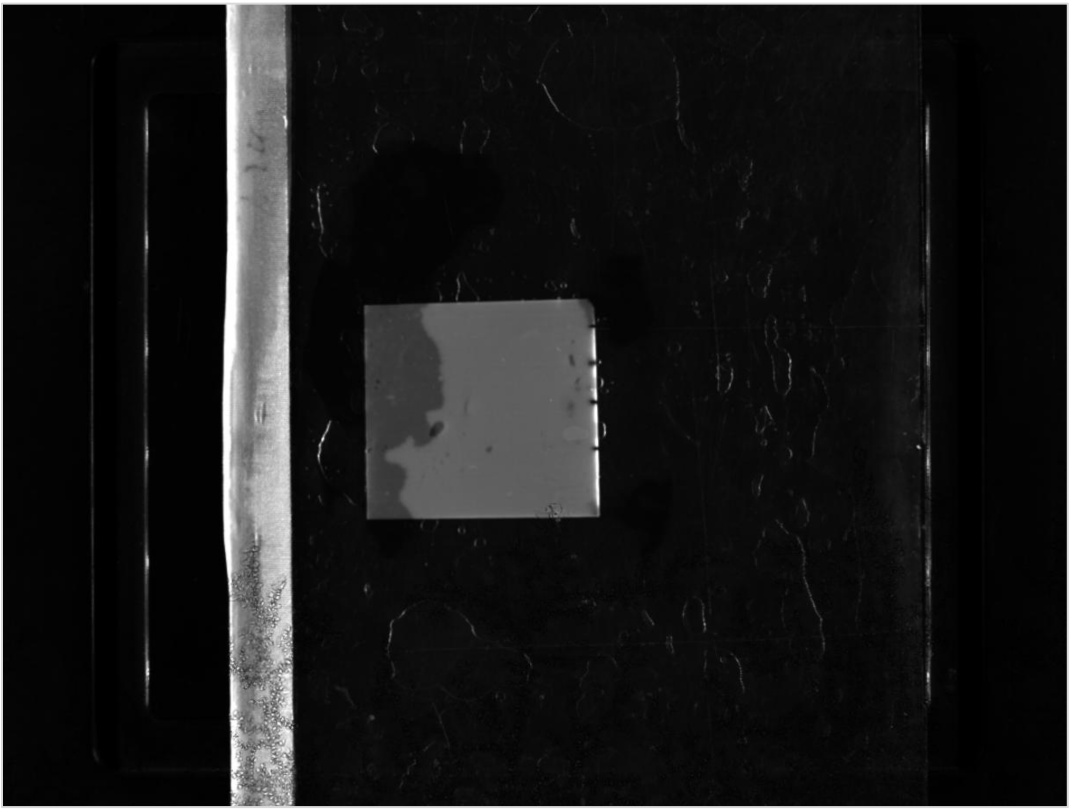

corresponding marker image

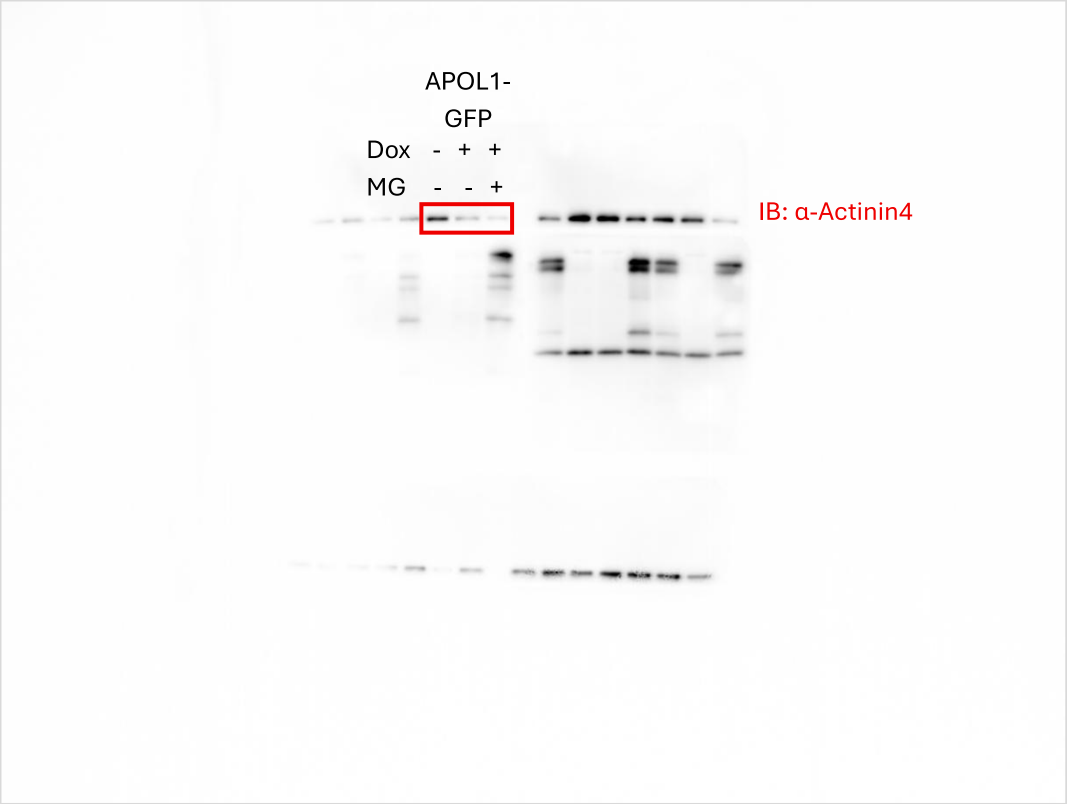

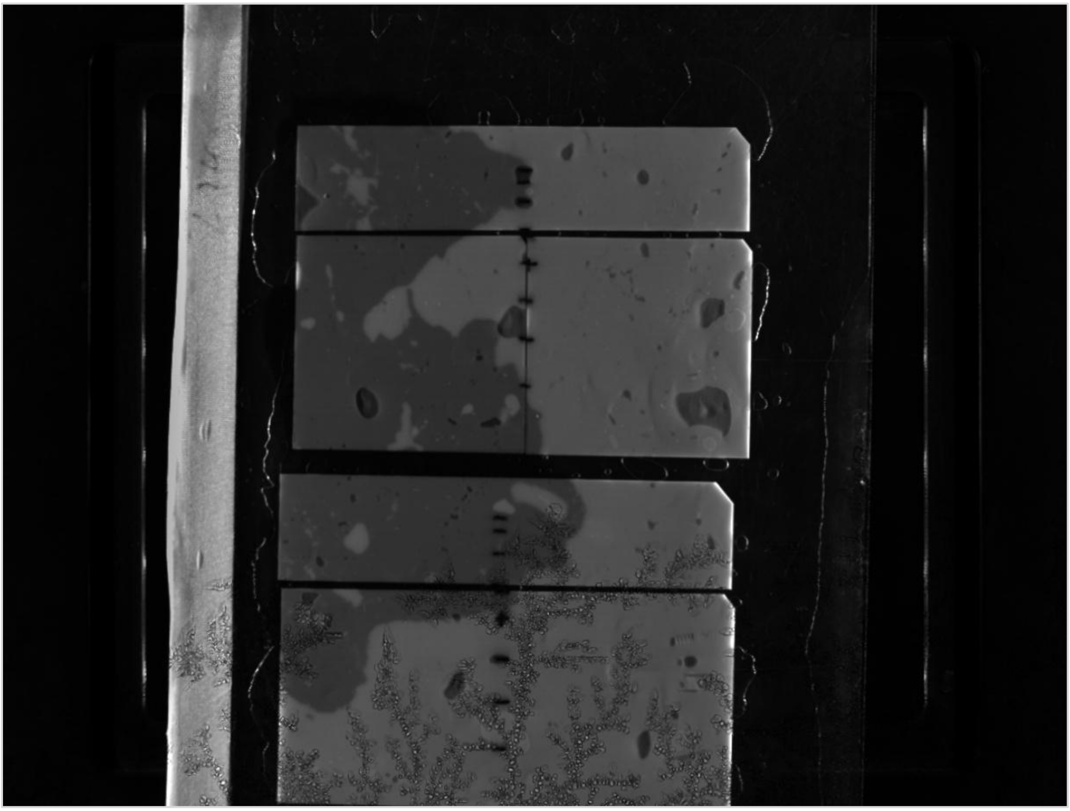

corresponding marker image

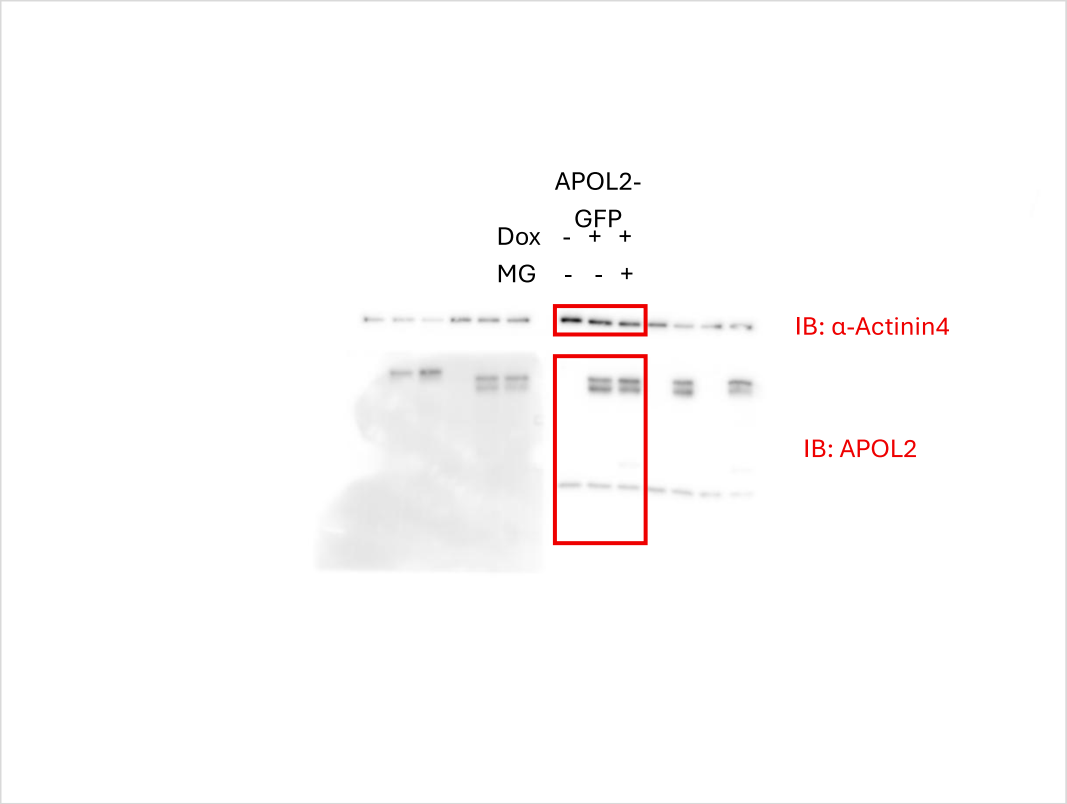

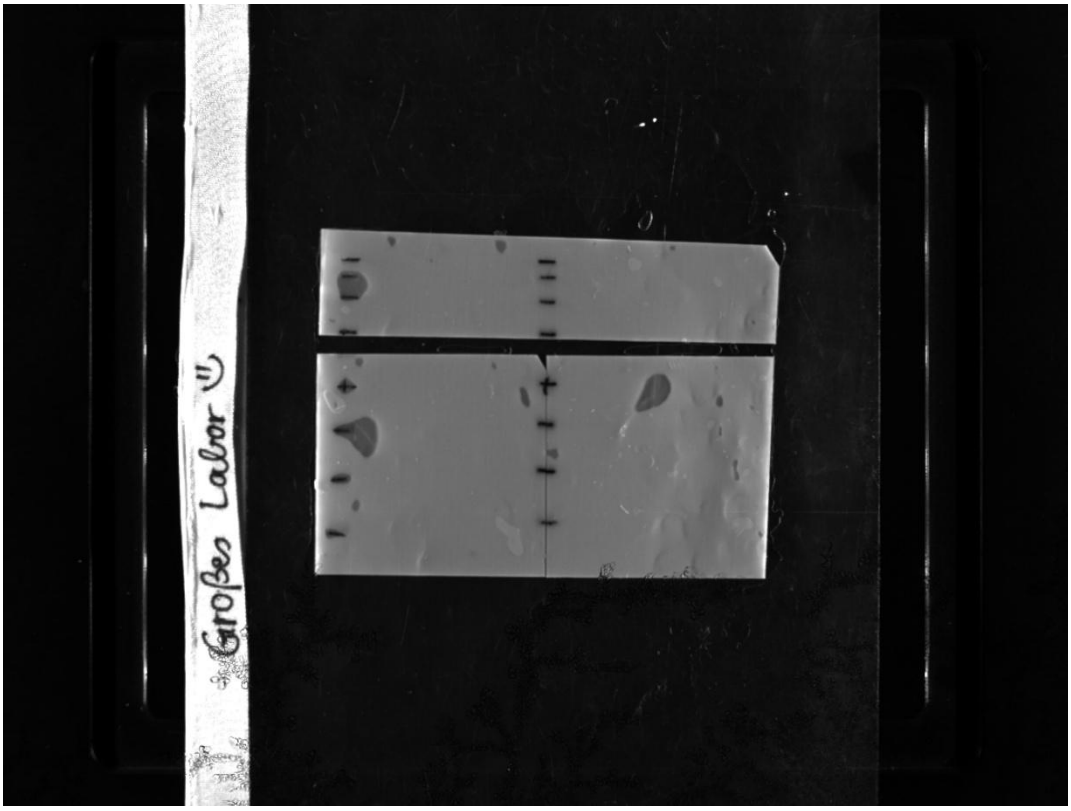

Corresponding marker image

Suppl. Figure SF2 B:

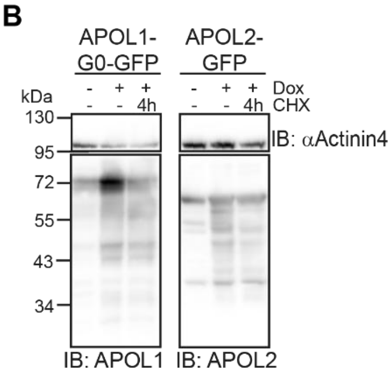

Suppl. Fig. SF2: *APOL1 and APOL2 show different degradation dynamics.*

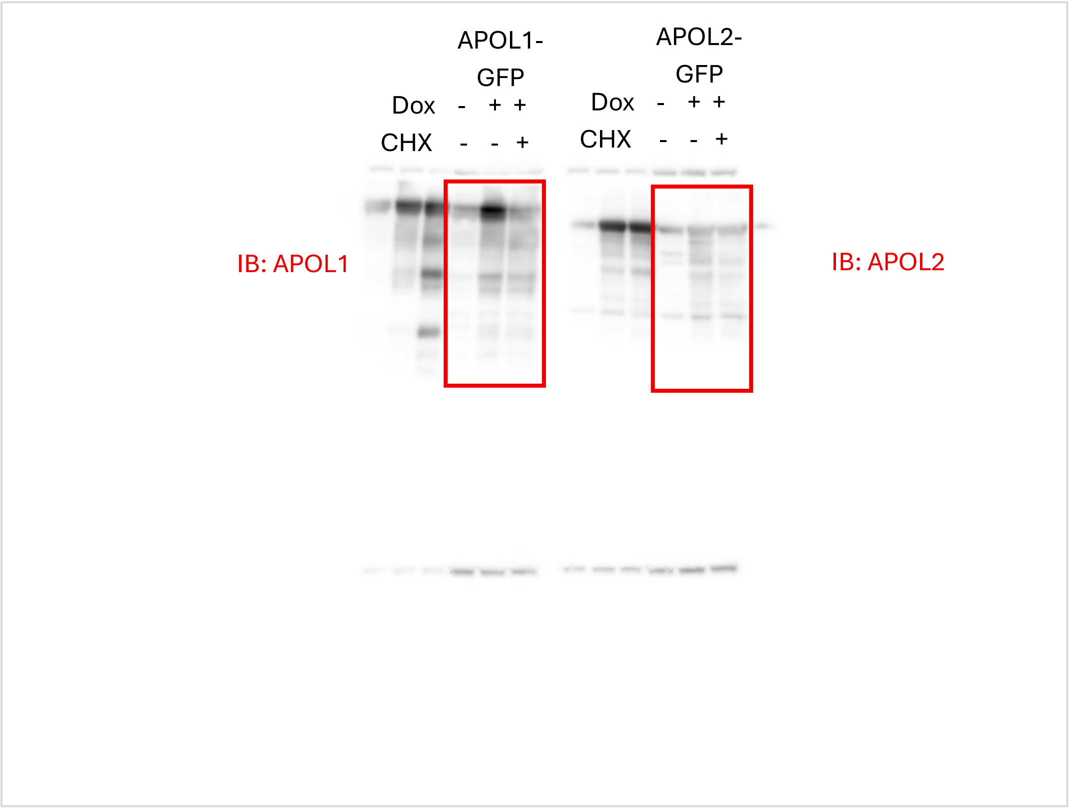

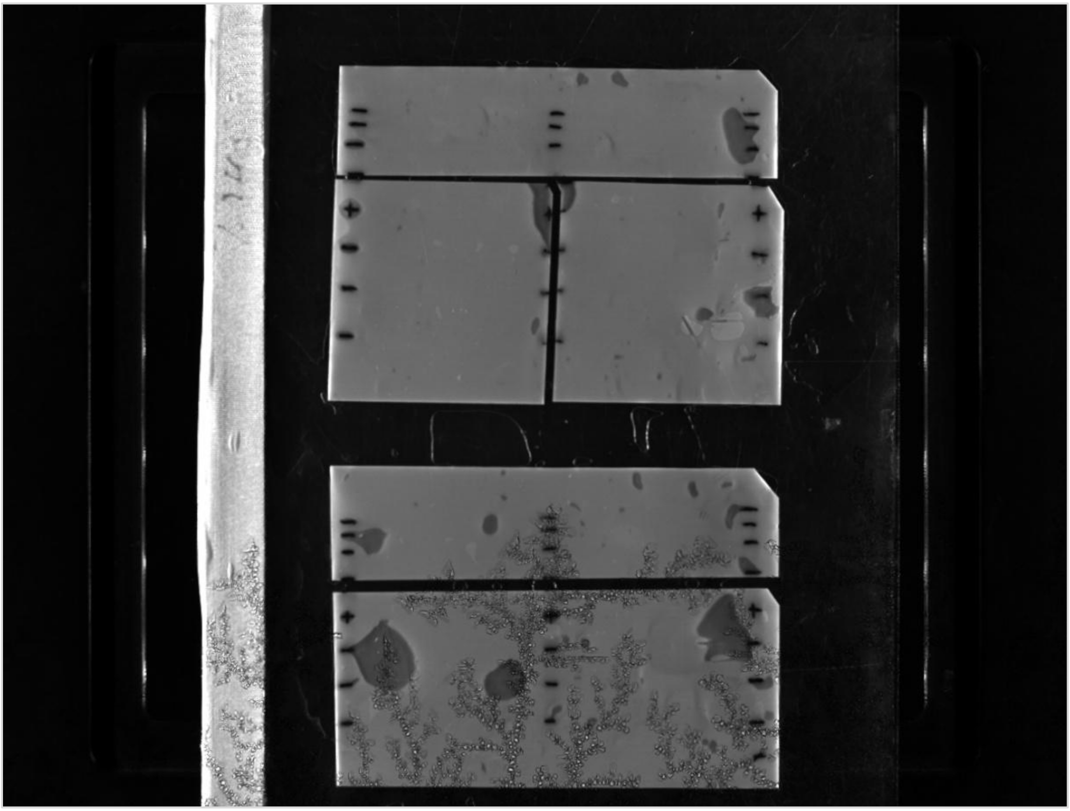

corresponding marker image

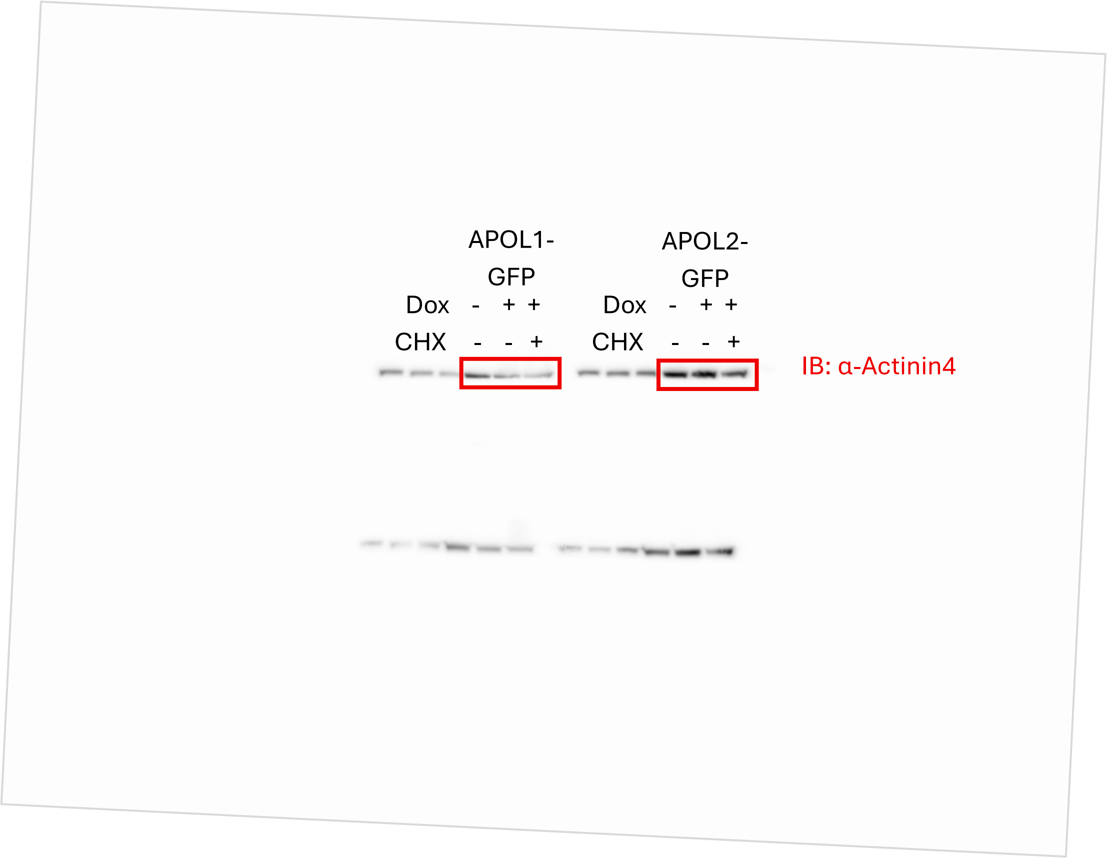

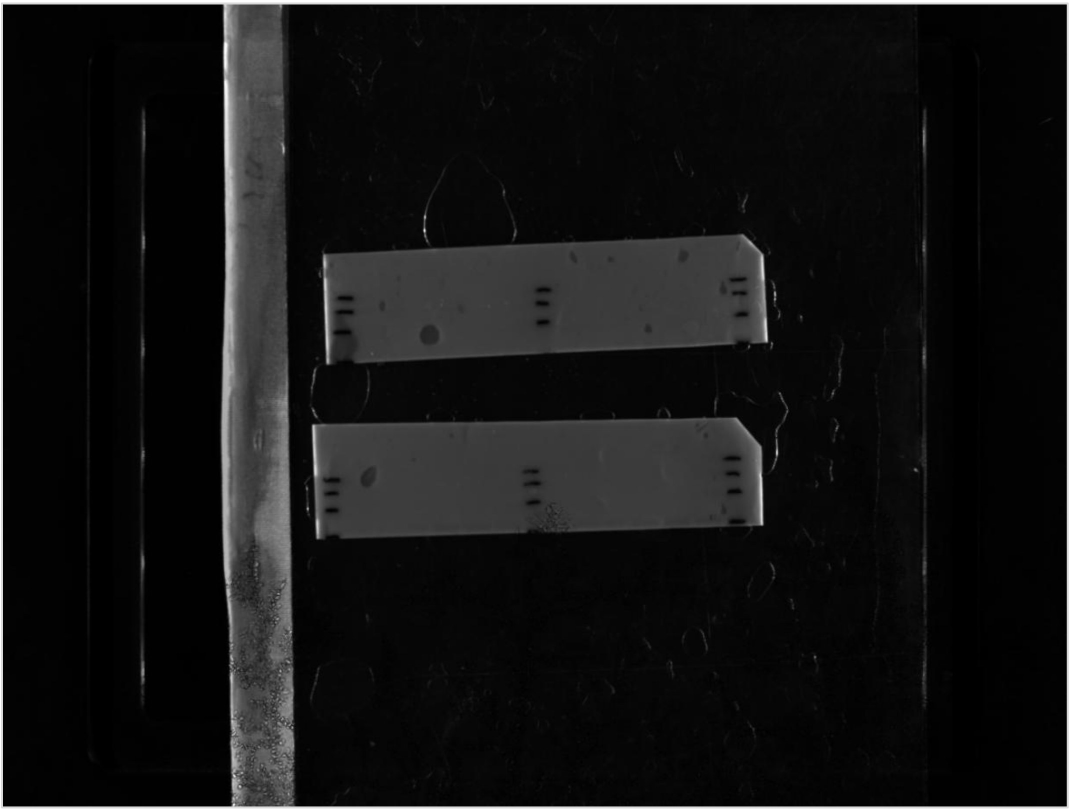

Corresponding marker image

Figure 4 B:

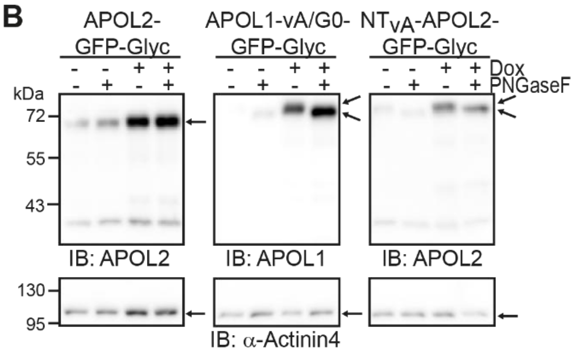

Fig. 4: *The N-terminal region of APOL1 contributes to proteasomal degradation.*

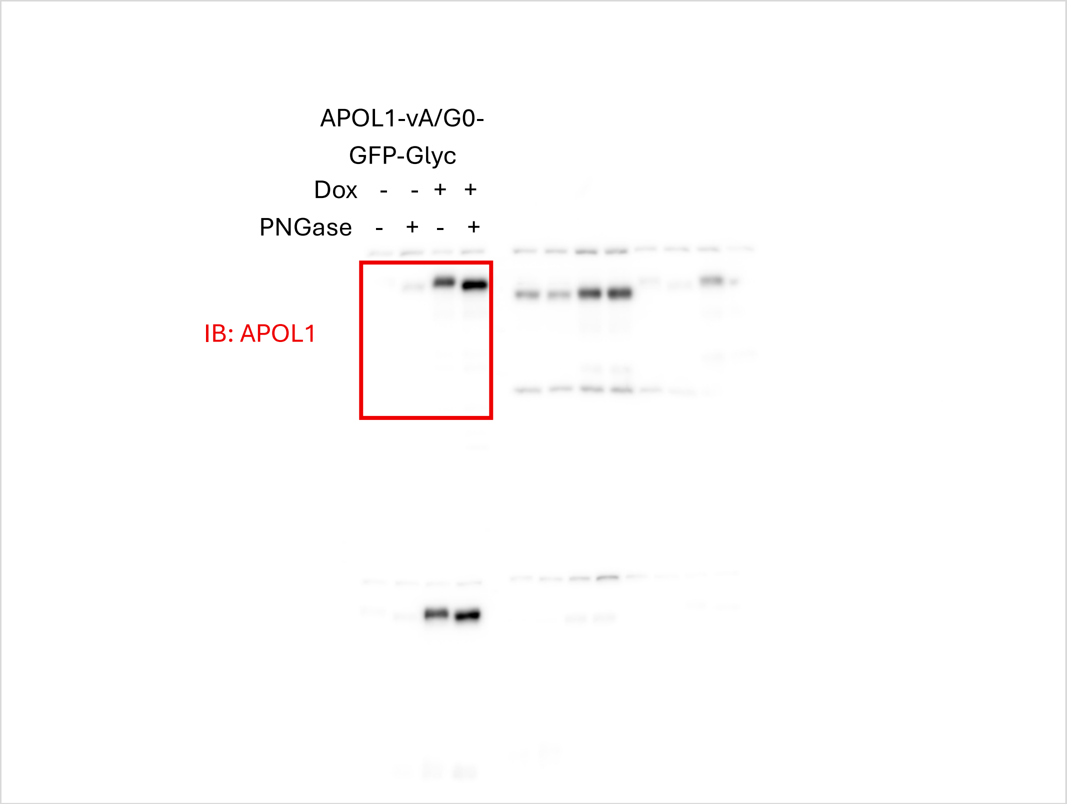
APOL1-vA/G0-
GFP-Glyc
Dox - - + +
PNGase - + - +
IB: APOL1

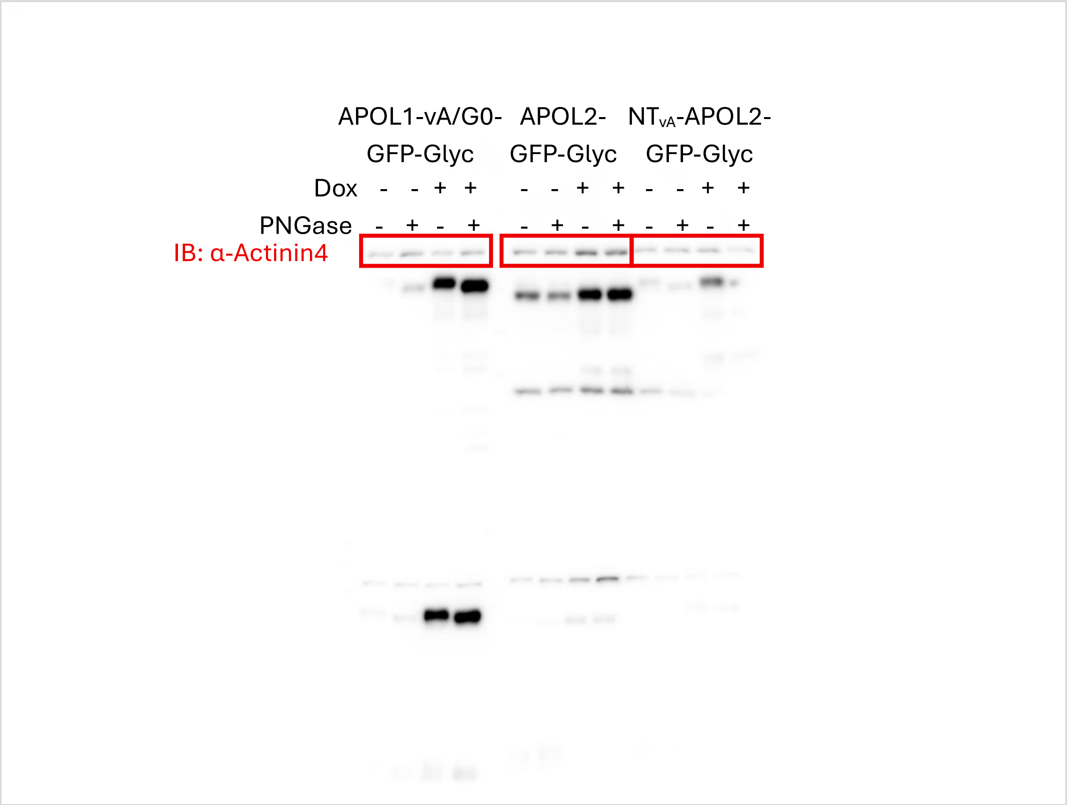
APOL1-vA/G0-
GFP-Glyc
APOL2-
GFP-Glyc
NT$_{vA}$-APOL2-
GFP-Glyc
Dox - - + + - - + + - - + +
PNGase - + - + - + - + - + - +
IB: α-Actinin4

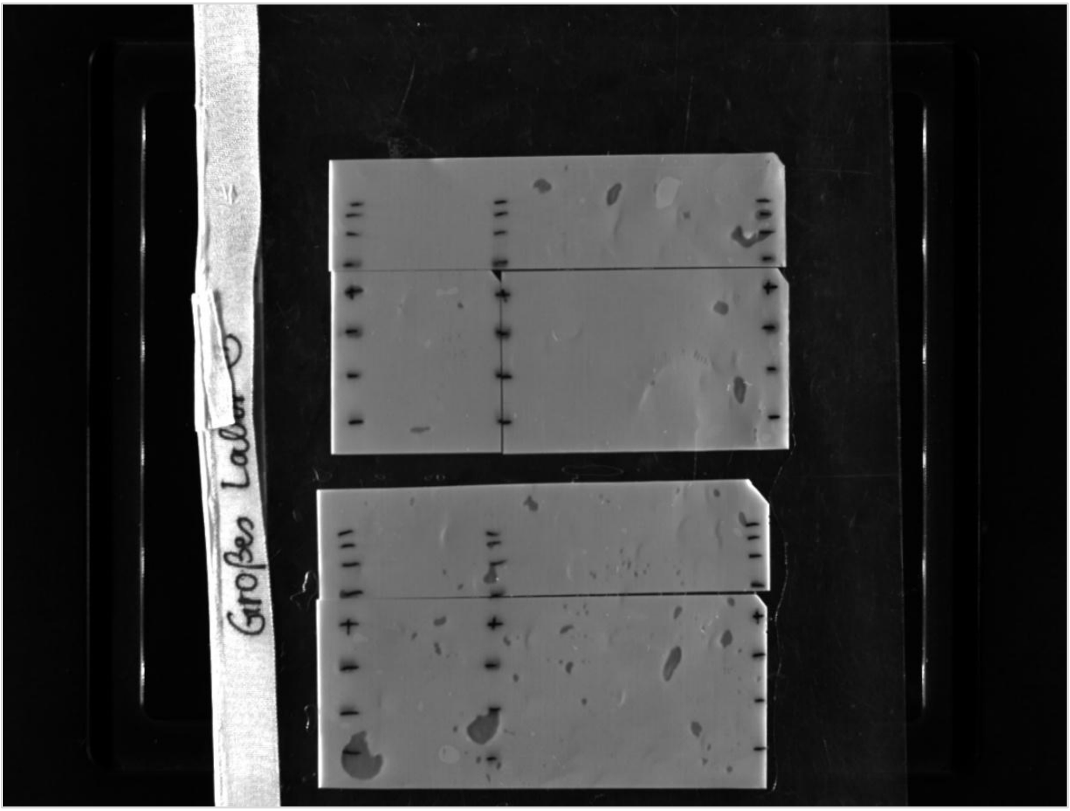

corresponding marker image

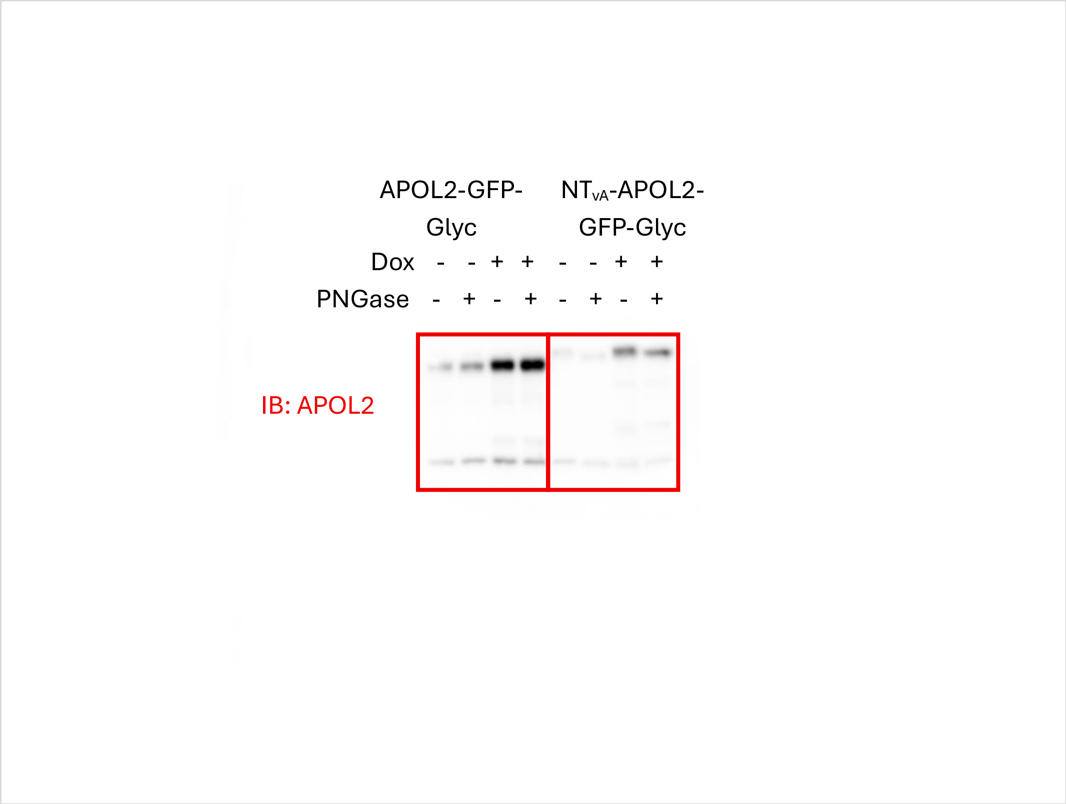

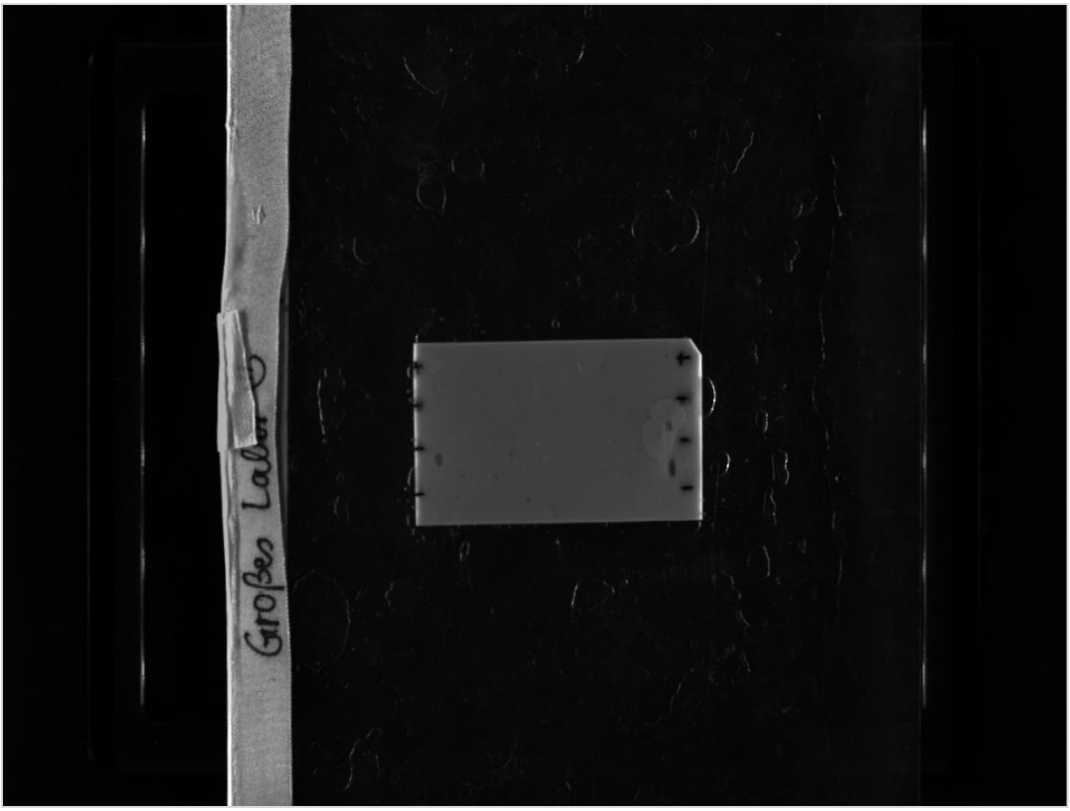

corresponding marker image
